# Additions to the cuckoo wasps (Hymenoptera, Chrysididae) of Mongolia, with description of eleven new species

**DOI:** 10.3897/zookeys.1068.73549

**Published:** 2021-11-08

**Authors:** Paolo Rosa, Maxim Yu. Proshchalykin, Marek Halada

**Affiliations:** 1 Laboratory of Zoology, University of Mons, Place du Parc 20, Mons, 7000, Belgium University of Mons Mons Belgium; 2 Federal Scientific Centre for East Asian Terrestrial Biodiversity, Far Eastern Branch of Russian Academy of Sciences, Vladivostok 690022, Russia Far Eastern Branch of Russian Academy of Sciences Vladivostok Russia; 3 Milady Horákové, 74, České Budějovice, 37012, Czech Republic Unaffiliated České Budějovice Czech Republic

**Keywords:** Taxonomy, new records, key, Central Asia, Palaearctic Region

## Abstract

An addendum to the recent checklist of the Chrysididae from Mongolia is given. Examination of old museum material and recently collected specimens has led to the discovery of eight new records for the country and eleven new species for science. Eight species are newly recorded from Mongolia: *Chrysisinclinata* Linsenmaier, 1959, *C.martinella* du Buysson, 1900, *C.speciosa* Radoszkowski, 1877, *Euchroeuspurpuratus* (Fabricius, 1787), *Holopygalucida* (Lepeletier, 1806), *H.similis* Mocsáry, 1889, *Hedychridiumfemoratum* (Dahlbom, 1854) and *H.leleji* Rosa, 2017. Two species, *Hedychridiumcupreum* (Dahlbom, 1845) and *H.propodeale* Rosa, 2017 are excluded from the checklist of Mongolian Chrysididae: the former is described here as *H.erythrosoma***sp. nov.**, the latter is identified as *H.leleji* Rosa, 2017. The hitherto unknown male of *Chrysismocsaryi* Radoszkowski, 1889 is described and illustrated. Eleven new species are described: *Chrysisstrakai***sp. nov.**, *C.woodi***sp. nov.**, *Hedychridiumerythrosoma***sp. nov.**, *H.frontale***sp. nov.**, *H.jacobsi***sp. nov.**, *H.splendens***sp. nov.**, *H.striatum***sp. nov.**, *H.varvarae***sp. nov.**, *H.weii***sp. nov.**, *Holopygatyrneri***sp. nov.**, and *Philoctetesboreki***sp. nov.** Keys to males and females of all known Mongolian species of *Hedychridium* Abeille de Perrin, 1878 are provided. The Mongolian cuckoo wasp fauna now comprises 107 species in 18 genera and two subfamilies.

## Introduction

Rosa et al. (2020) recently provided the first checklist of the Mongolian cuckoo wasps, including 90 species in 18 genera. The checklist was based on specimens collected by Czech entomologists (M. Halada, J. Halada, J. Straka and M. Kadlecová) in 2003–2007 and on the revision of published data, to clarify the confusion given in previous literature on true Mongolian localities. In fact, most of the published bibliographical data recorded for “Mongolia” actually refer to localities currently included in China (Inner Mongolia, Xinjiang, Gansu).

The present article is based on additional material collected by the same Czech entomologists and not included in the first checklist of the Mongolian cuckoo wasps. Further specimens were made available by Pavel Tyrner (Czech Republic) for this study. During our research, it was noticed that the cuckoo wasps, collected during the Soviet-Mongolian entomological expeditions in 1967–1982, were still unprepared and unidentified. The second author (MP) sorted out the unprepared specimens deposited at the Zoological Institute in St. Petersburg and isolated about 150 specimens to be studied for the next planned publications.

## Materials and methods

Terminology follows Lanes et al. (2020), Hymenoptera Anatomy Ontology (HAO 2021) and partly Kimsey and Bohart (1991). Abbreviations used in the descriptions are as follows: F, T and S are used for flagellomere, metasomal tergum and metasomal sternum, respectively; l/w=length/width; MOD = anterior ocellus diameter; MS = malar space, the shortest distance between base of mandible and lower margin of compound eye; OOL = the shortest distance between posterior ocellus and compound eye; P = pedicel; PD = puncture diameter; POL = the shortest distance between posterior ocelli. Other abbreviations used in the text: cat. (= catalogue), descr. (= description).

Pictures of the types were taken with a Nikon D700 connected to the microscope Togal SCZ and stacked with the software Combine ZP.

We have used the following abbreviations for collectors: JH – J. Halada; JS – J. Straka; MH – M. Halada; MK – M. Kadlecová; PT – Pavel Tyrner. An asterisk (*) marks the new records.

The holotypes of the newly-described species are deposited at the Zoological Institute, Russian Academy of Sciences, St. Petersburg (Russia) [ZIN] and at the Museum of Natural History of Milan (Italy) [Museo Civico di Storia Naturale, MSNM]; other types examined are deposited in the following Institutions and private collections: HNHM – Hungarian Natural History Museum, Budapest (Hungary); ISEA-PAS – Institute of Systematics and Evolution of Animals, Polish Academy of Sciences, Kraków (Poland); NHMW – Museum of Natural History, Vienna (Austria); MNHN – National Museum of Natural History, Paris (France); NMLS – Natur-Museum, Luzern (Switzerland); ZIN – Zoological Institute, Russian Academy of Sciences, St. Petersburg (Russia); ZMMU – Zoological Museum of Moscow Lomonosov State University (Russia); MHC – private collection of M. Halada (České Budějovice, Czech Republic); PRC – private collection of P. Rosa (Bernareggio, Italy); PTC – private collection of P. Tyrner (Litvínov, Czech Republic).

## Results

### Subfamily Chrysidinae

**Tribe Chrysidini**

**Genus *Chrysis* Linnaeus, 1761**

*Chrysis* Linnaeus, 1761: 414. Type species: *Sphexignita* Linnaeus, 1758 [= *Chrysisignita* (Linnaeus, 1758)], by subsequent designation of Latreille 1810: 437.

#### Chrysis inclinata

Taxon classificationAnimaliaHymenopteraChrysididae

Linsenmaier, 1959

5E16826D-91E3-5F4A-8AE8-8371564E1063

Figure 1A–E

Chrysis (Chrysis) inclinata Linsenmaier, 1959: 110. Holotype ♂; Greece: Corfu Is. (NMLS) (examined) (*succincta* group).

##### Material examined.

Mongolia: *Arkhangai*, 1 ♂, 90 km NE of Tsetserleg, 48°03'N; 102°25'E, 1400 m alt., 27.VII.2005, leg. JH (MHC).

##### Distribution.

*Mongolia (Arkhangai); Asiatic-European, known from Greece, south of former Yugoslavia (without precise locality) and Turkey (Linsenmaier 1959, 1968).

##### Remarks.

This is the most eastern record for *Chrysisinclinata*.

#### Chrysis martinella

Taxon classificationAnimaliaHymenopteraChrysididae

du Buysson, 1900

74E62FA4-BFC6-5B81-B0B2-C96BD6A68139

Chrysis martinella du Buysson, 1900: 142. Holotype ♀; Iran: Teheran (MNHN) (examined) (*aestiva* group).

##### Material examined.

Mongolia: *Tuv*, 1 ♂, Khangayn Mts, 5 km N of Khunt, 21.VII.2005, leg. P. Tyrner (PTC).

##### Distribution.

*Mongolia (Tuv); Asiatic-European, known from South-East Europe [not SW], Turkey and Caucasus (Rosa et al. 2019) and eastwards from Tajikistan (described as *C.martinellasolox* Semenov-Tian-Shanskij, 1954) to Afghanistan (described as *C.klapperichi* Balthasar, 1957).

##### Remarks.

The taxonomic treatment is given according to Kimsey & Bohart (1991), who placed several species and subspecies in synonymy of *Chrysismartinella*. The validity of these taxa is currently under revision.

**Figure 1.**
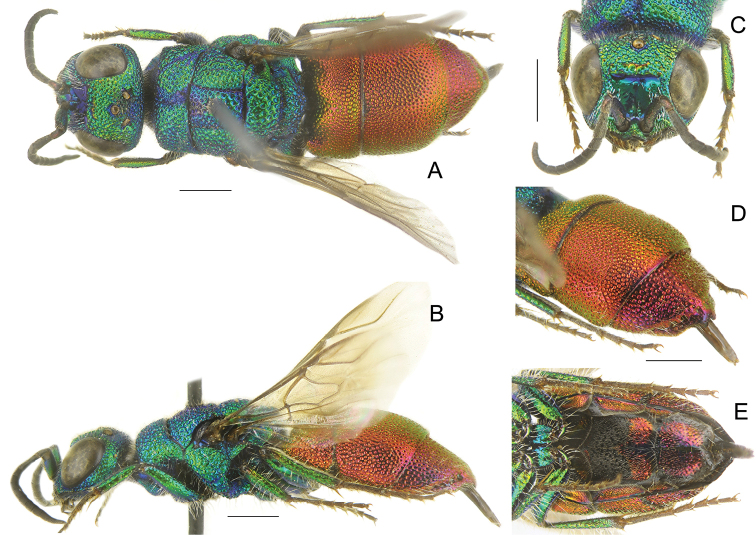
*Chrysisinclinata* Linsenmaier, female **A** habitus, dorsal view **B** habitus, lateral view **C** head, frontal view **D** metasoma, postero-lateral view **E** metasoma, ventral view. Scale bars: 1 mm.

#### Chrysis mocsaryi

Taxon classificationAnimaliaHymenopteraChrysididae

Radoszkowski, 1889

FA204426-CD14-5C89-9B02-BFFC420B8018

Figure 2A–G

Chrysis (Tetrachrysis) Mocsaryi Radoszkowski, 1889: 29. Holotype ♀; Mongolia: Kobden (Khovd) (ISEA-PAS) (examined) (*comparata* group). Mocsáry 1889: 426 (cat., descr., Mongolia).
Chrysis mocsaryi : Dalla Torre 1892: 78 (cat., Mongolia); Kimsey and Bohart 1991: 440 (cat., Mongolia: Kobden, comparata-scutellaris group); Rosa et al. 2015: 41 (cat., type series), 42 (fig. 4); 2020: 66 (cat.).

##### Material examined.

Mongolia: *Khovd*, 1 ♂, 20 km SE of Altaj, Elkhon, 26.VII.1970, leg. M. Kozlov (ZIN).

##### Diagnosis.

**Figure 2.**
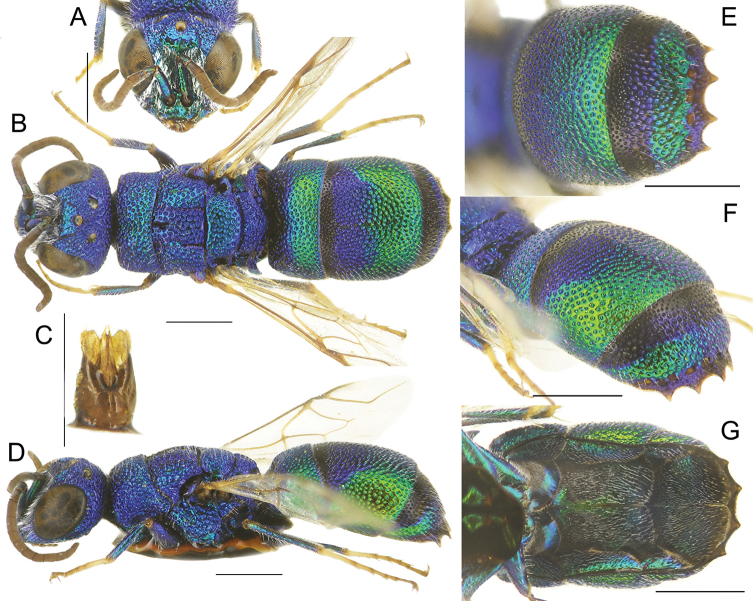
*Chrysismocsaryi* Radoszkowski, male **A** head, frontal view **B** habitus, dorsal view **C** genital capsule **D** habitus, lateral view **E** metasoma, posterior view **F** metasoma, postero-lateral view **G** metasoma, ventral view. Scale bars: 1 mm.

*Male* (hitherto unknown). Body length 6.7 mm. *Head*. Transverse frontal carina raised, with two lateral branches encircling the anterior ocellus (Fig. 2A and B); punctation in this area shallow to undefined; F1 as long as F2 and slightly metallic only basally; subantennal spaces elongate, 1.3 × MOD. *Mesosoma*. Anteromedial pronotal area widely depressed and anteromedian line indistinct (Fig. 2B); pronotum and mesonotum with even punctures, larger on the latter and polished interspaces; notauli as narrow, deep line; posterior propodeal projections narrow, apically acute and slightly divergent; mesopleuron with scrobal sulcus formed by wide, triangular and impunctate area; episternal sulcus deep and fully developed (Fig. 2D); fore wing with radial sector almost reaching wing margin; tarsi light yellow, meso- and meta-basitarsus whitish. *Metasoma*. Terga with dense punctures and polished interspaces (Fig. 2F); T1 dorsally, T2–T3 apicolaterally greenish to golden-greenish (possibly red in nature), contrasting with dark blue to black anteromedian area; apical margin of T3 blue after pit row; pits of pit row small, deep and rounded; apical margin quadridentate, with short, acute teeth (Fig. 2E); interval between median teeth slightly wider than interval between median and lateral tooth; metasomal longitudinal carina faint; black spots on S2 small, subrectangular, medially largely separated (Fig. 2G); genital capsule similar in structure to other species of the *C.scutellaris* group.

##### Distribution.

Mongolia (Khovd) (Radoszkowski 1889).

##### Remarks.

The specimen examined (Fig. 2A–G) belongs to the *scutellaris* species group and it is here considered as the unknown male of *Chrysismocsaryi*, based on the unusual metasomal colouration, similar to that of the female. Examination of more material is anyway needed to confirm this identification.

#### Chrysis speciosa

Taxon classificationAnimaliaHymenopteraChrysididae

Radoszkowski, 1877

243F96D1-DC2E-5F09-8645-8F0EB26839B0

Figure 3A–G

Chrysis speciosa Radoszkowski, 1877: 17. Lectotype ♂, designated by Bohart in Kimsey and Bohart 1991: 464; Uzbekistan: Tashkent desert (ZMMU) (examined) (maculicornis group).

##### Material examined.

Mongolia: *Dornogovi*, 5 ♂♂, 65 km SE of Chatan-Bulag, steppe, 1020 m alt., 2.VIII.2007, leg. MHMK (MHC, PRC); *Khovd*, 1 ♂, 15 km S of Bulgan, 29.VII.1970, leg. I. Kerzhner (ZIN).

##### Distribution.

*Mongolia (Dornogovi, Khovd); Tajikistan, Turkmenistan, Uzbekistan (Radoszkowski 1877; Mocsáry 1889; du Buysson in André 1896; Kimsey and Bohart 1991).

##### Remarks.

**Figure 3.**
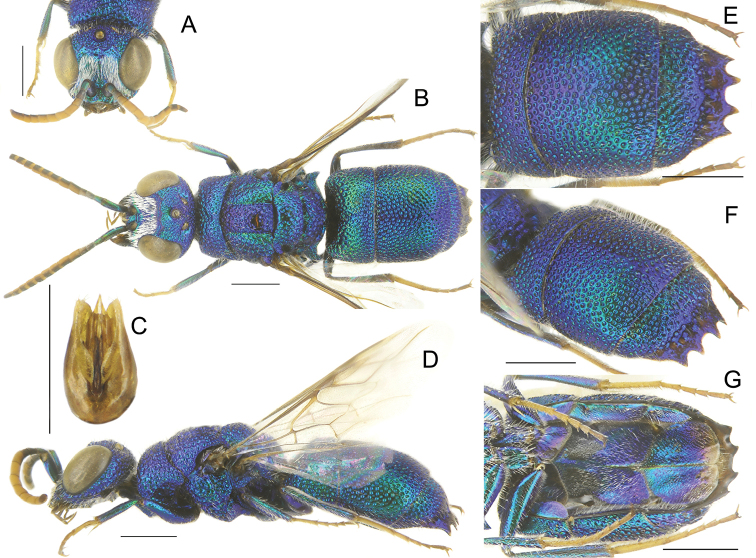
*Chrysisspeciosa* Radoszkowski, male **A** head, frontal view **B** habitus, dorsal view **C** genital capsule **D** habitus, lateral view **E** metasoma, posterior view **F** metasoma, postero-lateral view **G** metasoma, ventral view. Scale bars: 1 mm.

*Chrysisspeciosa* Radoszkowski, 1877 is a member of the *C.maculicornis* group and it is recognised by the colour pattern with body fully metallic blue; first and second flagellum short; flagellomeres extensively yellowish and tarsi yellow; metasoma with large, deep and even punctures (Fig. 3E); post pit row area on T3 wide; apical teeth on T3 elongate (Fig. 3D and F), with apex non-metallic brown (Fig. 3E and F); black spots on S2 large and subrectangular (Fig. 3G). Only two species with blue males are known in this group, *C.tatianae* Semenov-Tian-Shanskij, 1967 and *C.kokomerenica* Tarbinsky, 2002, both separated from *C.speciosa* by flagellomeres fully black.

Several Asiatic species are described in the *maculicornis* group, most of which are based on females only, with habitus and colouration similar to the common “*Chrysisdistincta* Mocsáry, 1887”: *C.contrasta* Tarbinsky, 2002; *C.fata* Tarbinsky, 2002; *C.kabulica* Balthasar, 1957; *C.semenovi* Radoszkowski, 1891; *C.subdistincta* Linsenmaier, 1968; *C.zarudniella* Semenov-Tian-Shanskij, 1967. Based on the copious Central Asian specimens deposited at ZIN, we can state that the males of some of these species, closely related to *C.disticta*, are entirely blue. Nevertheless, the correct attribution of the two sexes to the same species can be considered a challenge at this stage and without direct observation of copula in the field. Moreover, specimens collected in the same collecting event in Mongolia show large variation, although genitalia are rather similar.

#### Chrysis strakai

Taxon classificationAnimaliaHymenopteraChrysididae

Rosa, Proshchalykin & Halada sp. nov.

C145D01A-284D-567D-9DD6-24960AE681C2

http://zoobank.org/0A837FAE-D3E0-4881-A8C4-63F69EED8393

Figure 4A–G

##### Material examined.

***Holotype*:** ♂, Mongolia: *Bayankhongor*, 130 km S of Bayankhongor, 45°03'N; 100°59'E, 1240 m alt., Orog Nuur, 6–7.VII.2004, on saxaul, leg. JS (ZIN).

##### Diagnosis.

*Chrysisstrakai* sp. nov. is characterised by body colour metallic dark blue to violet with green and bluish reflections on metasoma. Face almost flat, with scapal basin, genae and clypeus laterally fully covered with long, appressed and silvery setae; transverse frontal carina faint; pronotum elongate with subparallel sides and deep, irregularly-sized punctures; mesonotum with sparse and polished interspaces; metasoma double punctate; T3 lateral margin deeply emarginated before lateral tooth; median teeth widely separated, with interval between median teeth almost twice as wide as interval between median and lateral tooth. The female is unknown.

##### Description.

**Figure 4.**
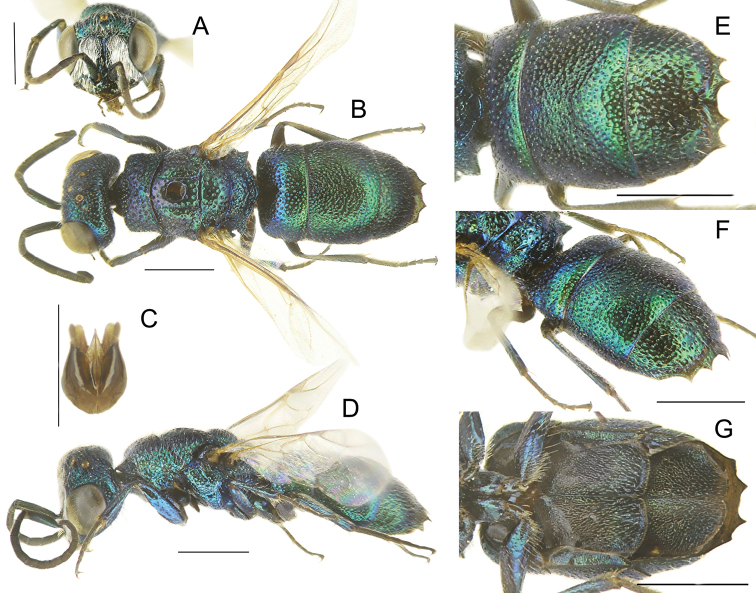
*Chrysisstrakai* sp. nov., male, holotype **A** head, frontal view **B** habitus, dorsal view **C** genital capsule **D** habitus, lateral view **E** metasoma, posterior view **F** metasoma, postero-lateral view **G** metasoma, ventral view. Scale bars: 1 mm.

*Male*. Body length 5.4 mm. *Head*. Vertex and brow with sparse, small punctures (about 0.2 × MOD), with tiny punctures on polished interspaces; brow with confluent punctures, forming radial pattern around anterior ocellus; depressed area in front of anterior ocellus and lateral to posterior ocelli; transverse frontal carina faint (Fig. 4A); in frontal view, uppermost margin of scapal basin edged, appearing as transverse carina; scapal basin flat densely micropunctate, with deep median line extended from uppermost margin of scapal basin to ¾ of scapal basin; scapal basin, excluding median line, genae and clypeus laterally fully covered with long, appressed and silvery setae; apical margin of clypeus triangular, non-metallic brown; mandible unidentate; genal carina developed from mid-eye to mandibular insertion, F1 as long as F2. OOL 2.5 × MOD; POL 2.0 × MOD; MS 1.1 × MOD; relative length of P:F1:F2:F3 = 1.0:1.3:1.3:0.9; subantennal space 1.4 × MOD. *Mesosoma*. Medial pronotal line unusually wide on anterior pronotal margin, as long as half-length of pronotum; pronotum coarsely punctate, with uneven sized punctures, denser and larger than those on mesonotum; interspaces with tiny punctures; mesoscutum with smaller, scattered and shallow punctures with wide interspaces (up to 3 PD); notauli as deep line, larger and triangular at base; lateral areas of mesoscutum with denser to sub-confluent punctures towards tegula; parapsidal signum hardly visible, as thin line amongst punctures; mesoscutellum with punctures similar to those on median area of mesoscutum, smaller and shallow punctate medially, denser laterally; metanotum with large, deep, irregular punctures mixed with smaller punctures, contiguous to confluent along the mesoscutellar-metanotal suture; posterior propodeal projections small, short, slightly divergent with straight posterior margin; posterior margin of metanotum with wide impunctate stripe (Fig. 4F); mesopleuron with scrobal sulcus wide, triangular and impunctate; episternal sulcus deep and fully developed only in the upper part of mesopleuron (Fig. 4D); forewing with radial sector complete, reaching wing margin and second radial cell closed. *Metasoma*. T1 with double punctation, punctures smaller than those on mesosoma and broadly separated with small punctures on interspaces; T2 dorsally with medium-sized, irregular punctures, deep and contiguous, obliquely engraved, well visible in posterior view (Fig. 4E); T3, with similar punctures; pits of pit row small, shallow and longitudinally elongate (Fig. 4E and F) separated to contiguous; T3 lateral margin deeply emarginated before apical, lateral tooth; apically with four short, pointed, triangular teeth (Fig. 4E and F); median teeth widely separated, with interval between median teeth almost twice as wide as interval between median and lateral tooth (Fig. 4F); metasomal terga without distinct median longitudinal carina; black spots on S2 large, medially separated, yet scarcely visible on the dark coloured sternum (Fig. 4G). *Colouration*. Body entirely metallic light blue with green reflections all over the body, on face, on bottom of mesosomal punctures, on lateral sides, on legs and sterna. Scape, pedicel and F1 light blue, other flagellomeres black. Wings clear, with brownish veins. *Vestiture*. Body with relatively short (1.0–1.5 × MOD) and whitish setae laterally.

*Female*. Unknown.

##### Etymology.

The specific epithet *strakai* (masculine noun in genitive) is dedicated to Jakub Straka (Prague, Czech Republic), who collected this undescribed species and other several new records for Mongolia, published in this article and in Rosa et al. (2020).

##### Comparative diagnosis.

*Chrysisstrakai* sp. nov. belongs to the *C.ehrenbergi* group. It cannot be confused with any other species known in the *C.ehrenbergi* group so far, based on its colouration, elongate pronotum and shape of T3.

##### Remarks.

Members of the *C.ehrenbergi* group usually show a red to golden-red colouration, which may turn into greenish in specimens preserved in collections. For this reason, based on a single specimen, we cannot exclude that the colouration of the holotype is based on a melanic specimen. However, the elongate shape of pronotum, the apical margin of T3 and genital capsule differentiate this species from the other few Central Asian species known so far.

##### Distribution.

Mongolia (Bayankhongor).

#### Chrysis woodi

Taxon classificationAnimaliaHymenopteraChrysididae

Rosa, Proshchalykin & Halada sp. nov.

CBF49CCC-0A15-5BC1-9FFC-F9B742C9F6C1

http://zoobank.org/2D1A5BFD-49BE-4569-80F7-5D3C7BEBEFFC

Figure 5A–G

##### Material examined.

***Holotype*:** ♂, Mongolia: *Dornogovi*, 65 km SE of Chatan-Bulag, 1020 m alt., 2.VIII.2007, leg. MH (MSNM).

##### Diagnosis.

*Chrysiswoodi* sp. nov. is characterised by the unusual colouration of flagellomeres, yellowish to brownish. Other relevant diagnostic characters are shape of the genital capsule, with different shape of gonocoxae before gonostylus; apical teeth on metasomal T3, aligned and almost subequal in length, with lateral ones slightly longer than the median pair; pits of the pit row on T3 deep, large or confluent; black spots on S2 large, medially fused, covering 2/3 of segment length.

##### Description.

**Figure 5.**
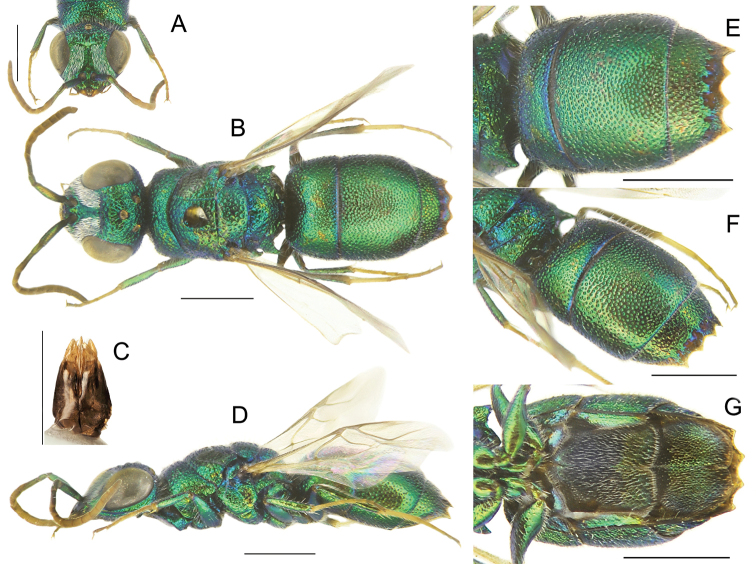
*Chrysiswoodi* sp. nov., male, holotype **A** head, frontal view **B** habitus, dorsal view **C** genital capsule **D** habitus, lateral view **E** metasoma, posterior view **F** metasoma, postero-lateral view **G** metasoma, ventral view. Scale bars: 1 mm.

*Male*. Body length 5.4 mm. *Head.* Scapal basin weakly concave with microridged median stripe, laterally micropunctate and covered with dense, short, silvery setae (Fig. 5A); brow prominent above scapal basin; uppermost area of the scapal basin distinctly polished; transverse frontal carina weak, straight, placed medially between anterior ocellus and uppermost margin of scapal basin; malar space elongate, 2.0 × MOD, shorter than F1; antenna thin and long, F1 5.0 × times as long as narrowest width; ventral side of F2–F3 lobulate (Fig. 5A and B); brow with confluent punctures, forming radial pattern around anterior ocellus; dense punctures on remaining part of vertex and ocellar area, with narrow and polished interspaces. OOL 1.5 × MOD; POL 2.3 × MOD; MS 2.0 × MOD; relative length of P:F1:F2:F3 = 1.0:1.7:1.1:0.8; subantennal space 1.1 × MOD. *Mesosoma.* Pronotum short, l/w: 3.3; medial pronotal line [= pronotal groove] short and shallow; pronotum with small punctures (PD ~ 0.2–0.3 × MOD) and micropunctate interspaces; median area of mesoscutum with shallow and small punctures (PD ~ 0.2–0.4 × MOD), with wide, polished interspaces (1-3 PD), denser postero-medially; interspaces mostly polished with few shallow dots; lateral areas of mesoscutum with similar punctation; punctures become denser along tegulae; mesoscutellum with slightly larger punctures posteriorly (PD ~ 0.5–0.6 × MOD) and small punctures on polished interspaces; anterolateral corners of scutellum above scutellar wing fossa expanded, subrectangular apically; metanotum with denser punctures; scutellar-metanotal suture deep, formed by longitudinally elongate foveae; posterior propodeal projections slightly divergent and basally concave; mesopleuron with wide scrobal sulcus and weak episternal sulcus, formed by small, aligned punctures in the upper part. Wings hyaline, with brown veins, tip of radial sector ending about 1.0 × MOD from anterior wing margin, leaving radial cell open. *Metasoma.* T1 with double punctation, with dense, small punctures (PD~ 0.2–0.3 × MOD) and micropunctate interspaces (Fig. 5F); T2–T3 with similar dense punctures, with polished intervals; pit row with deep, large and partially to fully confluent pits; apical margin of T3 with four short, triangular teeth, lateral ones slightly longer and more pointed (Fig. 5E and F); lateral edge of T3 straight; medial longitudinal carina faint; black spots on S2 elongate, covering about 2/3 of sternum length, medially fused, with posterior margin outcurved (Fig. 5G); apical margin of T3 bordered with narrow brownish, non-metallic rim; sterna covered with whitish short setae; genital capsule as in Fig. 5C, differently shaped compared with other species in this species group (Farhad et al. 2019). *Colouration*. Body entirely metallic green, with golden or rosy reflections on mesosoma and metasoma and blue pits of the pit row on T3; tegulae metallic green; mandibles light brown medially, metallic green at base. Scape, pedicel and large part of F1 metallic green, following flagellomeres yellowish ventrally and brownish dorsally (Fig. 5A, B and D); legs metallic green, tarsi yellowish, becoming darker distally; sterna metallic green.

*Female*. Unknown.

##### Etymology.

The specific epithet *woodi* (masculine noun in genitive) is dedicated to Thomas J. Wood (Mons, Belgium), for his continuous help in proofreading the manuscripts of our team.

##### Comparative diagnosis.

*Chrysiswoodi* sp. nov. belongs to the *C.varidens* group. Other two western Asian species show green colouration and similar habitus in this subgroup: *Chrysisbrunneamarginata* Farhad, Rosa & Talebi, 2019 (known from Iran) and *C.reperta* Vinokurov, 2010 (known from Kazakhstan). The first species is easily separable by shape of apical margin of T3, without metallic reflections and by shape of genital capsule (see Farhad et al. 2019). The second is separated by shape of apical margin of T3 with wavy median teeth and shape of black spots on S2 with straight posterior margin (vs. median apical teeth acute and arched posterior margin of black spots).

##### Remarks.

*Chrysisreperta* Vinokurov, 2010 was originally described with the name *C.repertus* and the name is here emended in *C.reperta*, being *repertus* a Latin masculine adjective not in accordance with the gender of the genus *Chrysis* Linnaeus, 1761.

##### Distribution.

Mongolia (Dornogovi).

**Genus *Euchroeus* Latreille, 1809**

*Euchroeus* Latreille, 1809: 49. Type species: *Chrysispurpurata* Fabricius, 1775, by monotypy.

#### Euchroeus purpuratus

Taxon classificationAnimaliaHymenopteraChrysididae

(Fabricius, 1787)

23C5EA36-2FD3-55D4-931E-AC720CD7E490

Chrysis purpurata Fabricius, 1787: 283. Neotype ♀ (designated by Pavesi & Strumia 1997: 195); France (Turin) (examined).

##### Material examined.

Mongolia: *Dornod*, 2 ♂♂, 20 km W of Choibalsan, 48°01'N; 114°14'E, 800 m alt., 24.VII.2007, leg. MH (MHC); *Khentii*, 1 ♂, 100 km NE of Ondorkhaan, Kerulen River, 970 m alt., 22.VII.2007, leg. JH (PRC); *Sukhbaatar*, 1 ♂, 100 km SSW of Baruun-Urt, 1100 m alt., 30.VII.2007, leg. MH (PRC).

##### Remarks.

The Mongolian population of *Euchroeuspurpuratus* is clearly separated from the western form by different punctation of clypeus, with small punctures and polished interspaces without micropunctation (vs. denser punctures, micropunctate on interspaces); elongate, spiniform process on propleuron and antero-ventrally on mesopleuron; metasoma mostly without darker bands. Future barcoding analysis will clarify the affinities between the western and Mongolian populations. Interestingly, another Mongolian species close to the genus *Euchroeus*, namely *Spinoliaspinosa* Rosa & Halada in Rosa et al. 2020, shares similar spines on propleuron.

##### Distribution.

*Mongolia (Dornod, Khentii, Sukhbaatar); West-Palaearctic from Western Europe to Central Asia (Rosa et al. 2019).

### Subfamily Chrysidinae

**Tribe Elampini**

**Genus *Hedychridium* Abeille de Perrin, 1878**

*Hedychridium* Abeille de Perrin, 1878: 3. Type species: *Hedychrumminutum* Lepeletier, 1806 [= *Hedychridiumardens* (Coquebert, 1801)], by subsequent designation of Ashmead 1902: 227.

#### Hedychridium belokobylskiji

Taxon classificationAnimaliaHymenopteraChrysididae

Rosa, 2017

4CEF3B05-5BCD-5961-9DC8-E54A8F2A10E8

Hedychridium belokobylskiji Rosa in Rosa et al. 2017a: 11. Holotype ♀; Russia: Eastern Siberia, Tyva Rep., 12 km SW of Samagaltai, Dyttyg-Khem River, 19.VII.2014, leg. A. Lelej, M. Proshchalykin, V. Loktionov (ZIN) (examined) (ardens group).

##### Material examined.

Mongolia: *Dornod*, 1 ♀, 20 km W of Choilbalsan, 800 m alt., 48°01'N; 114°14'E, 24.7.2007, leg. MH (PRC).

##### Distribution.

Mongolia (*Dornod, Tuv); Russia (Eastern Siberia) (Rosa et al. 2019, 2020).

#### Hedychridium erythrosoma

Taxon classificationAnimaliaHymenopteraChrysididae

Rosa, Proshchalykin & Halada sp. nov.

F38E7C84-6C29-5B5D-8E03-DCF332F7D3A9

http://zoobank.org/30D76669-7822-4225-A937-F72DEC5003A7

**Figure 6.**
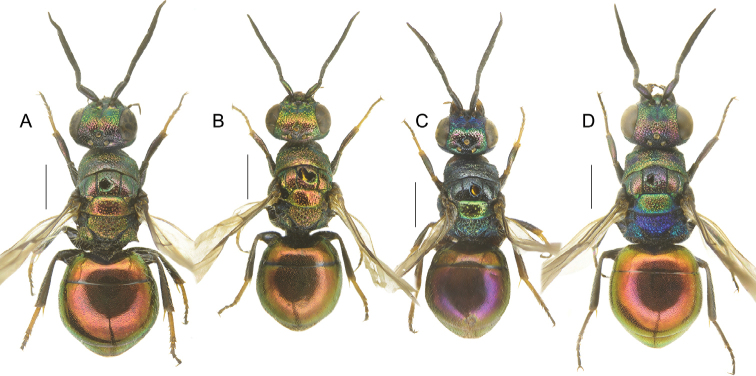
*Hedychridiumerythrosoma* sp. nov., female, holotype (**A**), male, paratype (**B**), *H.cupreum* (Dahlbom), female (from Estonia) (**C**), *H.asianum* Linsenmaier, female (from Mongolia) (**D**). Scale bars: 1 mm.

**Figure 7.**
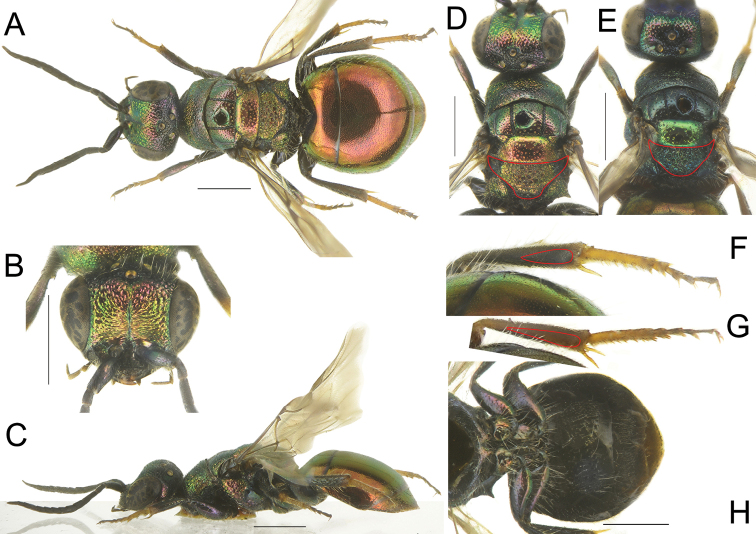
*Hedychridiumerythrosoma* sp. nov., female, holotype (**A–D, F, H**) and *H.cupreum* (Dahlbom), female (from Estonia) (**E, G**) **A** habitus, dorsal view **B** head, frontal view **C** habitus, lateral view **D** head and mesosoma, dorsal view **F** metaleg, right tibia and tarsi **H** metasoma, ventral view **E** head and mesosoma, dorsal view **G** metaleg, right tibia and tarsi. Scale bars: 1 mm.

**Figure 8.**
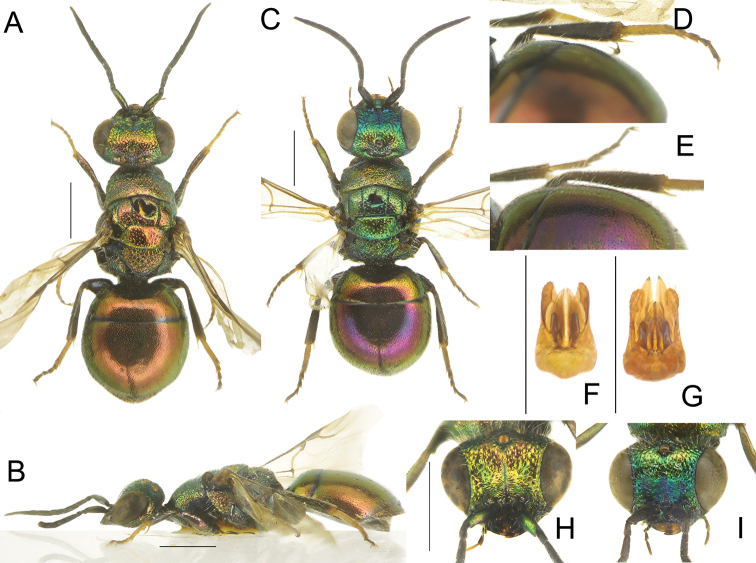
*Hedychridiumerythrosoma* sp. nov., male, paratype (**A, B, D, F, H**) and *H.cupreum* (Dahlbom), male (from Russia) (**C, E, G, I**) **A** habitus, dorsal view **B** habitus, lateral view **D** metaleg, right tibia and tarsi **F** genital capsule **H** head, frontal view **C** habitus, dorsal view **E** metaleg, right tibia and tarsi **G** genital capsule **I** head, frontal view. Scale bars: 1 mm.

Figures 6A, B , 7A–D, F, H , 8A, B, D, F and H

Hedychridium cupreum (Dahlbom, 1845): Rosa et al. 2020: 81 (cat., Mongolia: Bayankhongor, Dornogovi, Govi-Altai, Tuv, Umnugovi, Uvurkhangai, Zavkhan).

##### Material examined.

***Holotype*:** ♀, Mongolia: *Zavkhan*, 40 km SW of Uliastay, dunes, 18.VII.2005, leg. JH (ZIN). ***Paratypes***: *Zavkhan*, 1 ♀, 1 ♂, same data and locality of the holotype (PRC); *Tuv*, 1 ♂, 50 km E of Ulaanbaatar, Tuul River, 22.VI.2003, leg. JH (MHC); 1 ♀, Khangaun Mts., 5 km N of Khunt, 20.VII.2005, leg. JH (MHC); 1 ♀, 2 ♂♂, Khangaun Mts., 30 km S of Khunt, 20.VII.2005, leg. PT (PTC); 1♀, Khangaun Mts., 5 km N of Khunt, 21.VII.2005, leg. PT (PTC); 2 ♀♀, 50 km E of Ulaanbaatar, Tuul River, 22.VI.2003, leg. JH (MHC); *Govi-Altai*, 4 ♀♀, 70 km E of Altay City, Guulin, 14.VII.2005, leg. JH (MHC); *Bayankhongor*, 2 ♀♀, 16 km SW of Bayankhongor, 46°13'N; 100°30'E, 2165 m alt., 10.VII.2004, leg. JH (MHC); *Uvurkhangai*, 1♂, 12 km E of Aravaykheer, 46°22'N; 102°49'E, 1800 m alt., 3.VII.2004, leg. JH (MHC); *Zavkhan*, 1 ♀, 40 km SW of Uliastay, dunes, 18.VII.2005, leg. PT (PTC).

##### Diagnosis.

*Hedychridiumerythrosoma* sp. nov. is recognised by red body colour, with head and thorax coppery red, darker to violet on vertex, pronotum and mesoscutum; greenish on face and at sides of metasomal segments; metasomal sutures black; metasoma red flame dorsally, black ventrally; metanotum distinctly elongate, trapezoidal; metatibial [= hind tibial] spot short, impressed; male with distinct genital capsule.

##### Description.

*Female*. Body length 4.0–6.0 mm (holotype 5.7 mm). *Head.* Face with sharp ridges converging to mid-scapal basin (Fig. 7B); brow with large subcontiguous punctures, aligned towards anterior ocellus, with wide, polished interspaces; longitudinal mid-line deep, extending from anterior ocellus to clypeus; area in front of anterior ocellus and lateral to posterior ocelli depressed; ocellar triangle isosceles, with deep post-ocellar line; malar spaces short and impunctate; clypeus apically raised and bordered by thin, linear edge; mandibles bidentate and apically lighter from brownish to yellowish; setae on vertex thick, whitish and long (up to 2.0 × MOD). Relative length of P:F1:F2:F3 = 1.0:1.5:0.9:0.9; OOL = 2.2 × MOD; POL = 1.7 × MOD; MS = 0.4 × MOD. *Mesosoma.* Pronotum with large umbilicate punctures (Fig. 7A and B) and dense minute punctures on interspaces; mesonotum with sparse, shallow punctures, smaller than those on pronotum and not umbilicate, with sparse minute punctures on wide, polished interspaces; mesopleuron with dense punctures and polished mesepimeron; posterior propodeal projections small and acute, divergent; metatibia with impressed black spot covering about half of its length; metatarsomere 2 [= hind tarsal segment 2] shorter than metatarsomere 3; mesosoma and femora and tibiae with long whitish setae. *Metasoma*. T1–T3 with minute, even, shallow punctures and wide polished interspaces (2–3 x PD); posterior margin of T1–T2 with broad impunctate band (up to 1.0 × MOD); apical margin of T3 bordered by thin brownish rim; S2 with sparse, relatively large and deep punctures bearing long setae (up to 3.0 × MOD), without metallic reflections; metasoma laterally and posteriorly covered by long, thick and whitish setae. *Colouration.* Head and thorax red to coppery-red, darker to violet on vertex, pronotum and mesoscutum; greenish on face and at sides of metasomal segments; mesosomal sutures black; metasoma red flame dorsally, black ventrally; scape violet, pedicel shining black, flagellomeres black; tegulae non-metallic brown; femora and tibiae dark violet, tibial joints brownish; tarsi 1-2 yellowish, tarsi 3-5 brown; wing membrane slight infuscate. Body colouration more coppery-greenish in prepared (and photographed) specimens.

*Male.* Body length 5.0–5.5 mm. Similar to female in habitus and sculpture; main differences are: scapal basin with less sharp ridges (Fig. 8H) and with distinct lateral punctures; posterior propodeal projections smaller; genital capsule as in Fig. 8F.

##### Etymology.

The specific epithet *erythrosoma* derives from the Greek adjective *eruthros* (red) and the Greek noun *sōma* (body) and refers to the red body colouration of this species.

##### Comparative diagnosis.

We describe *Hedychridiumerythrosoma* sp. nov. in the *H.cupreum* species group. Species in this group are separated from those of the *H.ardens* group by the ridged scapal basin (punctate in *H.ardens* group), with ridges convergent to mid-face (transverse and parallel in *H.femoratum* group). In the previous checklist of the Mongolian cuckoo wasps, members of this taxon were identified as *H.cupreum* (Dahlbom, 1845), with a remark on their unusual colouration. Besides the red body colouration, *Hedychridiumerythrosoma* sp. nov. is separated from *H.cupreum* by shape of metanotum (see Fig. 7D and *H.cupreum* Fig. 7E), elongated medially in both sexes and metatibia black spot, shorter and deeply impressed (Fig. 7F) compared with the elongated and shallow pit of *H.cupreum* (Fig. 7G). Some paratypes lost their bright red colouration, which is partially turned into green. Males can be recognised by differently-shaped metanotum and shape of genital capsule (Fig. 8F), with different digitus and apex of gonocoxae.

##### Distribution.

Mongolia (Bayankhongor, Dornogovi, Govi-Altai, Tuv, Umnugovi, Uvurkhangai, Zavkhan).

#### Hedychridium femoratum

Taxon classificationAnimaliaHymenopteraChrysididae

(Dahlbom, 1854)

BE5F57FA-D5C6-54F0-B2D7-A9AF87E93793

Figure 9A–F

Hedychrum femoratum Dahlbom, 1854: 90. Holotype ♀; Austria (NHMW) (examined) (femoratum group).

##### Material examined.

Mongolia: *Dornod*, 1 ♀, 100 km W of Choibalsan, 820 m alt., 23.VII.2007, leg. M. Halada (PRC); *Dornogovi*, 2 ♀♀, 3 ♂♂, 65 km SE of Chatan-Bulag, 1020 m alt., 2.VIII.2007, leg. MH (PRC/MHC); 1 ♂, 2 km SE Khuvsgol, 5.VIII. 2007, leg. PT (PTC); *Tuv*, 1 ♂, 50 km N of Ulaanbaatar, E of Mandal, 1180 m alt., 8–13.VIII.2007, leg. MH (MHC).

##### Distribution.

*Mongolia (Dornod, Dornogovi, Tuv); from Europe to Turkey; Mongolia is the easternmost record for this species.

**Figure 9.**
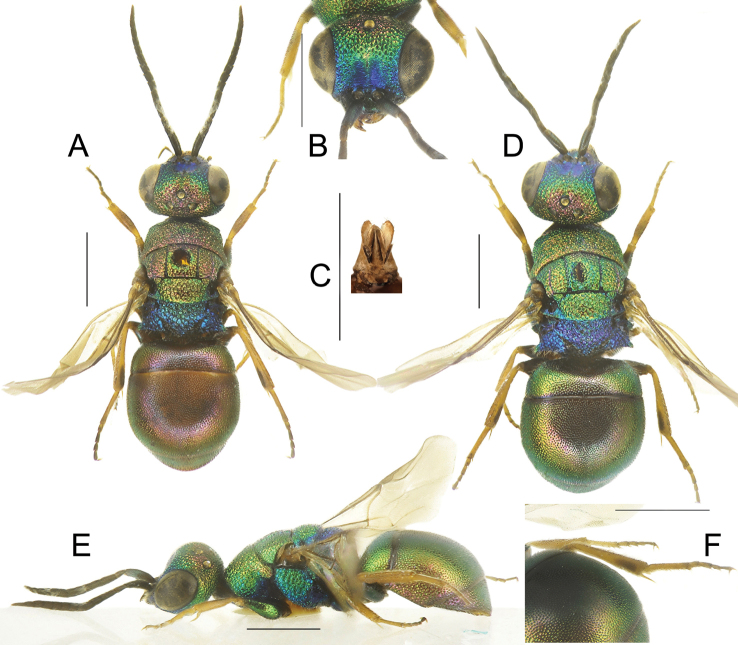
*Hedychridiumfemoratum* (Dahlbom), female (**A**) and male (**B–F**) (from Mongolia) **A** habitus, dorsal view **B** head, frontal view **C** genital capsule **D** habitus, dorsal view **E** habitus, lateral view **F** metaleg, right tibia and tarsi. Scale bars: 1 mm.

#### Hedychridium frontale

Taxon classificationAnimaliaHymenopteraChrysididae

Rosa, Proshchalykin & Halada sp. nov.

E1403960-5899-58B4-9C18-6873A54FB55B

http://zoobank.org/D08AE934-0800-419B-AC0F-917757F63147

**Figure 10.**
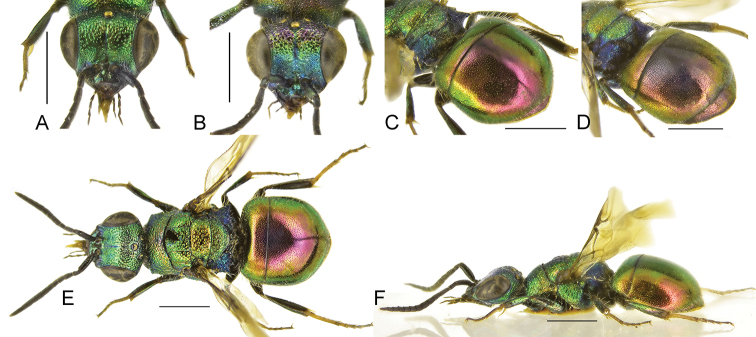
*Hedychridiumfrontale* sp. nov., female, paratype (**A, C, E, F**) and *H.ardens* (Coquebert), female (from Mongolia) (**B, D**) **A** head, frontal view **C** metasoma, postero-lateral view **E** habitus, dorsal view **F** habitus, lateral view **B** head, frontal view **D** metasoma, postero-lateral view. Scale bars: 1 mm.

Figures 10A, C, E and F

##### Material examined.

***Holotype*:** ♀, Mongolia: *Bayankhongor*, 86 km NW of Bayankhongor, 46°50'N; 100°04'E, 2070 m alt., 13–15.VII.2004, leg. JS (MSNM). ***Paratype***: 1 ♀, *Govi-Altai*, 70 km E of Altay City, Guulin, 14.VII.2005, leg. PT (PTC).

##### Diagnosis.

*Hedychridiumfrontale* sp. nov. is characterised by wide, polished interspaces on brow; scapal basin finely microridged medially only on lower half, from mid-face to clypeus; clypeal apical margin bordered by a thick brownish rim 3 × MOD long; head concolorous green, with a bluish highlight laterally to clypeus; S2 without metallic spot.

##### Description.

*Female*. Body length 4.5–5.0 mm (holotype 4.7 mm). *Head.* Face flat, brow convex above scapal basin (Fig. 10A); brow with small (0.3 × MOD) punctures, with wide, polished interspaces (1-3 × PD); scapal basin finely, transversely microridged in the lower half, close to clypeus; face micropunctate laterally and with short, appressed, whitish setae close to clypeus; longitudinal mid-line complete from frons to clypeus; vertex with small, dense punctures; area in front of anterior ocellus and lateral to posterior ocelli depressed; ocellar triangle isosceles, without ocellar line; malar spaces micropunctate; clypeus apically bordered by slightly arcuate, elongate (3 × MOD), brown thickening; mandibles bidentate, yet subapical tooth blunt; mouthparts elongate; vertex and sides with short, sparse whitish setae. Relative length of P:F1:F2:F3 = 1.0:1.1:0.8:0.7; OOL = 2.0 × MOD; POL = 2.1 × MOD; MS = 1.0 × MOD. *Mesosoma.* Pronotum narrowed anteriorly and with sharp edge on anterior margin; coarse, irregular, umbilicate punctures of different size, with polished to corrugated interspaces and small punctures on interspaces; mesonotum with wide interspaces, wider on lateral areas; punctures slightly larger at base of mesoscutum and on mesoscutellum, with scattered small punctures; mesopleuron with dense punctures of different size, without polished interspaces; posterior propodeal projections acute, divergent; median area of metapostnotum (median area of postnotum situated between the propodeum and T1) triangular, narrower than in other species of *H.ardens* group; metatibia flat, with black spot covering almost its full length; metatarsomere 2 slightly longer than metatarsomere 3; pro-, mesopleuron and femora with long whitish setae. *Metasoma*. T1–T3 with minute, punctures; punctures slightly denser antero-dorsally on T2; large punctures mixed to minute punctures laterally; punctation overall with wide, polished interspaces; apical margin of T3 bordered by a relatively wide hyaline rim (0.25 × MOD); S2 with sparse, minute punctures bearing long setae (up to 3 × MOD), without metallic spot. *Colouration.* Head metallic green, with bluish reflections close to clypeus; propodeum blue; metasoma golden laterally and red to purplish dorsally; scape, pedicel and flagellum black; tegulae non-metallic brown; femora and tibiae green on outer side, black to dark violet in ventral view; tarsi brownish, darker distally; wing membrane infuscate.

*Male.* Unknown.

##### Etymology.

The specific epithet *frontale* derives from the Latin adjective *frontalis* (forehead) and refers to the different sculpture and colouration of this species compared with the common and widespread *H.ardens*.

##### Comparative diagnosis.

We describe *Hedychridiumfrontale* sp. nov. in the *Hedychridiumardens* species group. This species is closely related to *Hedychridiumardens* (Coquebert, 1801), yet some diagnostic morphological characters clearly separate it. Brow more convex above scapal basin; different sculpture, with wide, polished interspaces (Fig. 10A) [vs. densely punctate in *H.ardens* (Fig. 10B)]; scapal basin medially, finely microridged only on lower half, from mid-face to clypeus (vs. microridged area longitudinally more expanded); lateral micropunctures from mid- eye to malar space (vs. more extended); clypeal apical margin bordered by a thick brownish rim 3 × MOD long (vs. not longer than 2 × MOD); head concolorous green, with a bluish highlight laterally to clypeus (vs. bicolour with purplish vertex, greenish brow and blue face); mouthparts more elongate; median area of metapostnotum (median area of postnotum situated between the propodeum and T1) smaller (compare Fig. 10C and D); metasomal with sparser punctures (Fig. 10C); S2 without metallic spot (vs. with metallic spot); legs black to dark purple in ventral view (vs. greenish).

##### Distribution.

Mongolia (Bayankhongor, Govi-Altai).

#### Hedychridium jacobsi

Taxon classificationAnimaliaHymenopteraChrysididae

Rosa, Proshchalykin & Halada sp. nov.

C136EC10-C792-58E5-90A0-B531B40F2601

http://zoobank.org/BDB431D3-826F-49E6-805C-96620CD89A8C

Figure 11A–G

##### Material examined.

***Holotype*:** ♂, Mongolia: *Dornogovi*, 28 km SE of Chatan-Bulag, 2.VIII.2007, leg. MH (ZIN). ***Paratypes***: 6 ♀♀, 3 ♂♂, same data and locality of holotype, leg. MH, leg. PT (MHC, PRC, PTC); 1 ♀, 3 ♂♂, 65 km SE of Chatan-Bulag, 1020 m alt., 2.VIII.2007, leg. MH (MHC); *Bayankhongor*, 1 ♀, 3 ♂♂, 75 km S of Bayankhongor, 45°20'N; 100°48.5'E, 1330 m alt., 8–9.VII.2004, leg. JH, JS (MHC, PRC).

##### Diagnosis.

*Hedychridiumjacobsi* sp. nov. is characterised by legs and F1 yellowish, also in the male; F1 elongate (l/w = 3.0 in female, 4.0 in male); sculpture of scapal basin with sharp transverse ridges covering almost all face in frontal view; ridges on scapal basin may produce darkened to black effect on scapal basin when examined at different angles.

##### Description.

**Figure 11.**
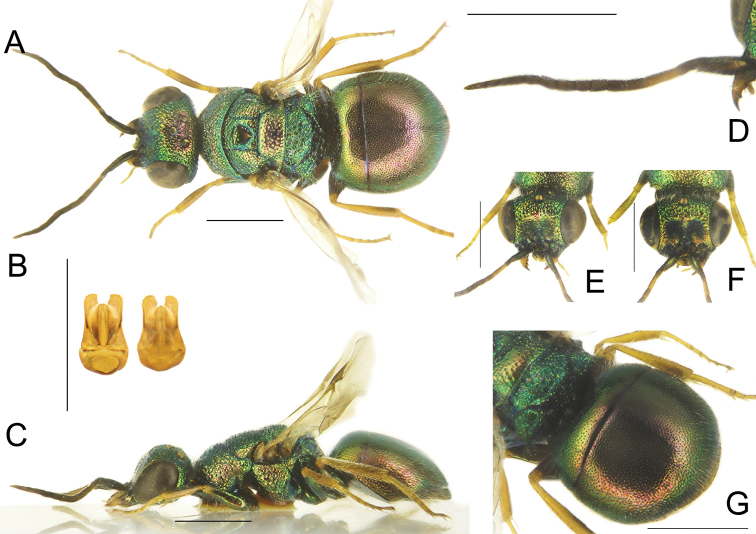
*Hedychridiumjacobsi* sp. nov., male, holotype (**A–E, G**); male, paratype (**F**) **A** habitus, dorsal view **B** genital capsule, ventral and dorsal view **C** habitus, lateral view **D** right antenna, lateral view **E** head, frontal view **F** head, frontal view; **G** metasoma, postero-lateral view. Scale bars: 1 mm.

*Male*. Body length 4.0–4.5 mm (holotype 4.1 mm). *Head.* Face with scapal basin slightly deep; scapal basin with sharp, transverse ridges, almost reaching eye margin (Fig. 11E); laterally with sparse punctures amongst ridges and single row of large punctures between facial ridges and eye margin; longitudinal mid-line incomplete, distinctly visible from upper scapal basin to clypeus; with large, subcontiguous punctures on brow and small punctures on vertex and temples; area in front of anterior ocellus and lateral to posterior ocelli depressed; ocellar triangle isosceles, without ocellar line; malar spaces micropunctate; clypeus apico-medially bordered by thin, brown rim; mandibles bidentate; mouthparts short, not exceeding the mandibles. Relative length of P:F1:F2:F3 = 1.0:1.6:1.0:0.7; OOL = 1.7 × MOD; POL = 2.0 × MOD; MS = 0.6 × MOD. *Mesosoma.* Pronotum with irregular punctures of different size, somewhere contiguous and with polished to corrugated interspaces; mesonotum with small punctures and wide interspaces, somewhere corrugated; punctures slightly larger at base of mesoscutum and distinctly larger at sides of mesoscutellum, medially with scattered punctures; posterior propodeal projections acute, divergent; metatibia with depression on inner side, as long as half of its length and only partially darkened; light brown metatarsomere 2 shorter than metatarsomere 3; pro-, mesopleuron and femora with long whitish setae. *Metasoma*. T1–T3 with minute, even and dense punctures on all surface; S2 with sparse, minute punctures bearing long setae; with large violet spot, covering about half segment; apical margin of T3 bordered by thin hyaline rim; genital capsule as in Fig. 11B. *Colouration.* Head and mesosoma metallic green with bronze to violet reflections dorsally; metasoma with rosy to violet reflections (possibly metallic red in nature); scape and pedicel bronze, F1 yellowish (Fig. 11D); F2 brownish; rest of flagellum brown; tegulae non-metallic yellowish; femora joints yellowish, tibiae yellowish with slight greenish reflections on outer side of fore tibia; tarsi yellowish, brownish distally; wing membrane clear, nervures light brown.

*Female.* Body length 3.5–4.0. Similar to male in habitus and body sculpture; F1 slightly shorter than male, yellowish; spot on second metasomal sternum golden.

*Paratypes*. Males show variability in antennae colour, with F1–F2 pale yellowish; distribution of dark, black area amongst ridges on face (Fig. 11F); punctures on pronotum, more or less spaced with polished interspaces; shape of posterior propodeal processes, more or less spiny rather than triangular.

##### Etymology.

The specific epithet *jacobsi* (masculine noun in genitive) is dedicated to Maarten Jacobs (Herentals, Belgium), for his contribution to the study of Chrysididae with superb images taken in nature and for providing some Mongolian specimens from his past naturalistic trips in the country.

##### Comparative diagnosis.

We describe *Hedychridiumjacobsi* sp. nov. in the *Hedychridiumfemoratum* species group. It is related to *H.femoratum* and other species in this group for its general habitus, yellowish legs and F1. It is separated from other species of this group by elongate F1 (l/w = 3.0 in female, 4.0 in male) (Fig. 11D); yellowish F1 in male (brown like other flagellomeres in other species); unique sculpture of scapal basin with sharp transverse ridges covering almost all face in frontal view; ridges on scapal basin produce a unique darkened to black effect on scapal basin, when examined at different angles (Fig. 11 E and F).

##### Distribution.

Mongolia (Bayankhongor, Dornogovi).

#### Hedychridium leleji

Taxon classificationAnimaliaHymenopteraChrysididae

Rosa, 2017

B0102D17-35AF-5A80-9AD6-81968B484327

Figure 12 A–F

Hedychridium leleji Rosa in Rosa et al. 2017a: 23. Holotype ♀; Russia: Eastern Siberia, Tyva Rep., 30 km W Erzin, Yamaalyg, 4.VII.2013, leg. V. Loktionov & M. Proshchalykin (ZIN) (examined).

##### Material examined.

Mongolia: *Govi-Altai*, 1 ♂, 70 km E of Altay city, Guulin, 14.VII.2005, leg. JH (PRC). 1 ♀, same data and locality, leg. PT (PTC).

##### Distribution.

*Mongolia (Govi-Altai); Russia (Eastern Siberia) (Rosa et al. 2019).

##### Remarks.

This specimen was previously identified as *Hedychridiumpropodeale* Rosa, 2017 (Rosa et al. 2019). After the re-preparation and re-examination of the specimen, the correction of the previous identification was needed.

**Figure 12.**
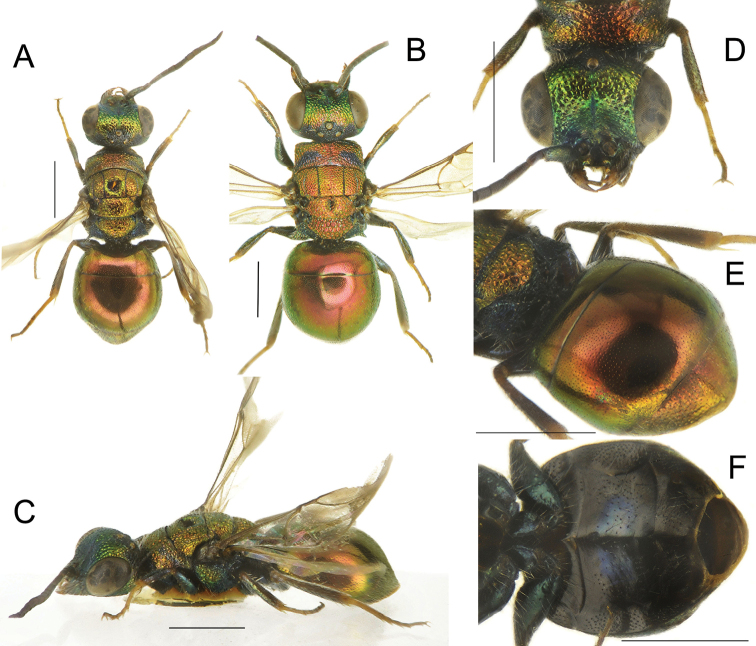
*Hedychridiumleleji* Rosa, females (from Mongolia, **A, C–F**) and (from Siberia **B**) **A, B** habitus, dorsal view **C** habitus, lateral view **D** head, frontal view **E** metasoma, postero-lateral view **F** metasoma, ventral view. Scale bars: 1 mm.

#### Hedychridium splendens

Taxon classificationAnimaliaHymenopteraChrysididae

Rosa, Proshchalykin & Halada sp. nov.

EF1271A4-E6B1-57A0-8421-86B01ACFFFD2

http://zoobank.org/DDB9B14A-4E03-4CDE-A29D-EC372FB6F828

Figure 13A–F

##### Material examined.

***Holotype*:** ♀, Mongolia: *Uvurkhangai*, 159 km of SW Aravaykheer, 45°11'N; 101°26'E, 1250 m alt., 5.VII.2004, leg. JH (MSNM).

##### Diagnosis.

*Hedychridiumspendens* sp. nov. is characterised by sparse and shallow body punctures with wide, polished interspaces, which generate a shining effect; legs and F1 yellowish, F1 short (l/w = 2.0); metatibia with small, brownish spot; S2 with wide, bronze spot.

##### Description.

**Figure 13.**
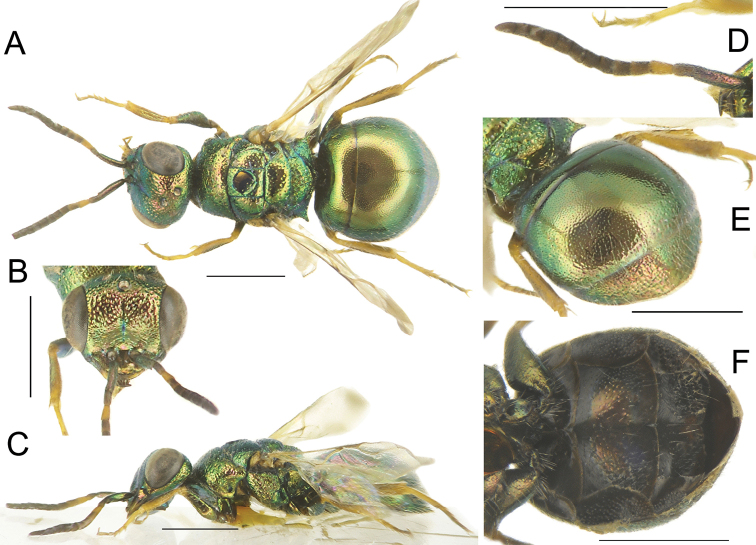
*Hedychridiumsplendens* sp. nov., female, holotype **A** habitus, dorsal view **B** head, frontal view **C** habitus, lateral view **D** right antenna, lateral view **E** metasoma, postero-lateral view **F** metasoma, ventral view. Scale bars: 1 mm.

*Female*. Body length 4.1 mm. *Head.* Face almost flat; scapal basin slightly incised medially, with longitudinal mid-line extended from anterior ocellus to lower scapal basin; scapal basin finely transversely microridged (Fig. 13B); brow with small, shallow punctures, with wide, polished interspaces; posterior ocelli with lateral area depressed and polished; ocellar triangle isosceles, without ocellar line; malar spaces micropunctate; clypeus apically bordered by thin, brown thickening; mouthparts slightly elongate. Relative length of P:F1:F2:F3 = 1.0:1.3:1.0:0.8; OOL = 1.8 × MOD; POL = 2.6 × MOD; MS = 0.8 × MOD. *Mesosoma.* Pronotum with small, deep punctures and shallow minute punctures on interspaces; mesonotum with scattered, shallow punctures, with few minute punctures on interspaces; mesopleuron with denser, larger punctures and with small punctures on interspaces, rugose anteriorly; posterior propodeal projections acute, divergent; metatibia with relatively small brown spot covering about half of its length; metatarsomere 2 a little shorter than metatarsomere 3; femora with long whitish setae. *Metasoma*. T1–T2 with minute, shallow punctures; punctures denser antero-dorsally on T1–T2; T3 with denser punctures and rugose interspaces; apical margin of T3 bordered by thin brownish rim, with long (up to 2.0 × MOD), whitish setae laterally and postero-laterally; S2 with sparse punctures bearing long setae, with antero-median coppery spot, covering more than half segment. *Colouration.* Body entirely shining green with coppery reflections; scape and pedicel coppery, F1 yellowish; F2 brownish (Fig. 13D); rest of flagellum brown; tegulae non-metallic yellowish; femora green on outer side; tibiae darker with opalescent reflections on outer side; tibial joints yellowish; tarsi 1-2 yellowish, tarsi 3-5 brownish; wing membrane clear, nervures light brown.

*Male.* Unknown.

##### Etymology.

The specific epithet is derived from the Latin *splendens* (shining), present participle of the verb splendeō, which refers to the shining body of this cuckoo wasp, due to sparse, small and shallow punctures, with wide polished interspaces.

##### Comparative diagnosis.

We describe *Hedychridiumspendens* sp. nov. in the *H.femoratum* species group and it is related to *H.jacobsi* by its general habitus, F1 and yellowish legs. However, it is separated from the latter by unmodified sculpture of the face, with scapal basin only finely, transversally microridged (vs. sharp transverse ridges covering almost all face); shorter F1, l/w = 2.0 (l/w = 3.0 in female, 4.0 in male); sparse and shallow body punctation with wide, polished interspaces (vs. punctation denser).

##### Distribution.

Mongolia (Uvurkhangai).

#### Hedychridium striatum

Taxon classificationAnimaliaHymenopteraChrysididae

Rosa, Proshchalykin & Halada sp. nov.

FD82000E-07E0-541A-B91D-CA1F8579FD07

http://zoobank.org/FC6A1BD5-0DEE-4CBA-ABA0-077EDCDCD99D

Figure 14A–F

##### Material examined.

***Holotype*:** ♀, Mongolia, *Tuv*, 75 km W of Ulaanbaatar, dunes, 2.VIII.2005, leg. JH (ZIN). ***Paratypes***: 3 ♀♀, 3 ♂♂, same date and locality as holotype (MHC, PRC); *Govi-Altai*, 1 ♀, 1 ♂, 70 km E of Altay City, Guulin, 14.VII.2005, leg. JH (MHC); 4 ♀♀, 8 ♂♂, same data and locality, leg. PT (PTC).

##### Diagnosis.

*Hedychridiumstriatum* sp. nov. is characterised by transversal (on mesoscutum), longitudinal (on mesoscutellum) and oblique (on mesopleuron) wrinkles (more evident in male); propodeal posterior projections divergent and spiniform; legs largely yellowish; metasoma scattered punctures, with wide polished interspaces; S2 extensively metallic.

##### Description.

**Figure 14.**
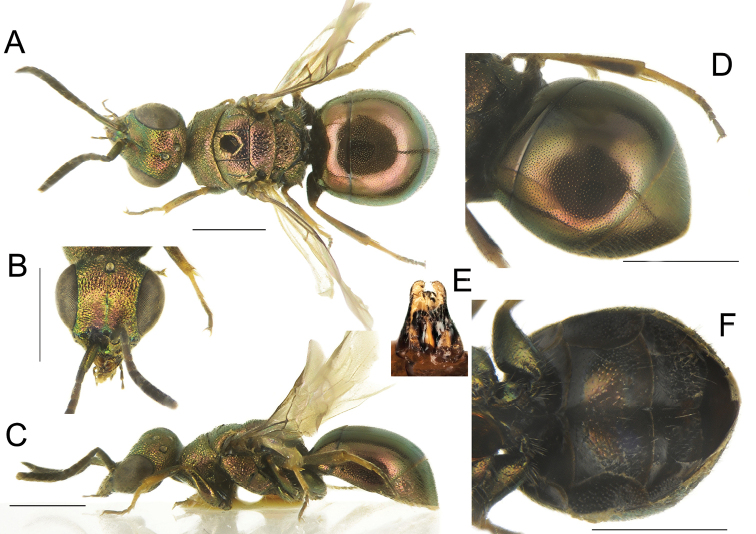
*Hedychridiumstriatum* sp. nov., female, holotype (**A–D, F**) and male, paratype (**E**) **A** habitus, dorsal view **B** head, frontal view **C** habitus, lateral view **D** metasoma, postero-lateral view **E** genital capsule **F** metasoma, ventral view. Scale bars: 1 mm.

*Female*. Body length 4.0–4.5 mm (holotype 4.5 mm). *Head.* Face nearly flat, with narrow and elongate eyes (Fig. 14B); brow between vertex and scapal basin with contiguous, longitudinally aligned punctures; scapal basin transversally microridged; incomplete longitudinal mid-line extended from anterior ocellus almost to clypeus; at sides, between scapal basin and eye, with small punctures transversally aligned amongst fine wrinkles; vertex with deep punctures and polished interspaces; clypeus elongate, subantennal space about 1.5 × MOD; ocellar triangle isosceles, with deep ocellar line connecting posterior ocelli. Relative length of P:F1:F2:F3 = 1.0:1.6:1.0:0.8; OOL = 2.4 × MOD; POL = 1.8 × MOD; MS = 1.0 × MOD. *Mesosoma.* Pronotum with coarse, contiguous punctures; mesoscutum with sparse, shallow punctures amongst transverse wrinkles; mesoscutellum with sparse punctures amongst longitudinal wrinkles; mesopleuron with similar punctures and oblique wrinkles. Posterior propodeal projections divergent and spiniform (Fig. 14A); mid-tibia with small, oval, darkened area; metatibia with large triangular black area as long as half of its length. *Metasoma.* T1–T3 with sparse, minute and even punctures, equally spaced (2-3 PD), T1 smooth along median line and posteriorly; T3 with narrow brownish rim on posterior margin; S2 with metallic coppery spot (Fig. 14E). *Colouration.* Body dorsally metallic red-bronze; scape and pedicel greenish, flagellum black; tegulae bronze; femora bronze; tibiae yellowish, outer side slightly bronze to opalescent, tibial joints yellowish; tarsi 1-2 yellowish, tarsi 3-5 dark brown.

*Male.* Body length 3.5–4.0 mm. Similar to female in habitus, colour pattern and unusual sculpture, yet face unmodified, whereas in female, looks narrow and elongate; genital capsule as in Fig. 14E, triangularly shaped, narrowed apically.

##### Etymology.

The specific epithet *striatum* derives from the Latin adjective *striatus*, *a*, *um* (striated) and refers to the unusual striated sculpture on mesosoma.

##### Comparative diagnosis.

We tentatively describe *Hedychridiumstriatum* sp. nov. in the *Hedychridiumardens* species group; nevertheless, for some diagnostic characters, such as genital capsule and yellowish legs, it can be confused with members of other species group (*H.rhodinum* and *H.femoratum* groups); the female shows narrow and elongate eyes as members of the *H.planifrons* group. This species is anyway easily recognisable from all other *Hedychridium* species by its unique mesonotal sculpture with punctures amongst transversal wrinkles on mesoscutum, longitudinal wrinkles on mesoscutellum and oblique wrinkles on mesopleuron (more evident in male); propodeal posterior projections divergent and spiniform; legs largely yellowish; metasoma with scattered punctures, with wide polished interspaces.

##### Distribution.

Mongolia (Govi-Altai, Tuv).

#### Hedychridium varvarae

Taxon classificationAnimaliaHymenopteraChrysididae

Rosa, Proshchalykin & Halada sp. nov.

9D060DDA-1034-572F-AE2B-F744BC37FFBE

http://zoobank.org/DB76F413-F24B-4B24-ABD7-1E1F7AA7F98F

Figure 15A–F

##### Material examined.

***Holotype*:** ♀, Mongolia, *Dornogovi*, 28 km SE of Chatan-Bulag, 3.VIII.2007, leg. MH (MSNM).

##### Diagnosis.

*Hedychridiumvarvarae* sp. nov. is characterised by yellowish F1; uniform fore body green colouration, including propodeum and face; metasoma green to reddish once dehydrated (in nature, presumably red); brow with wide, polished interspaces; pronotum narrowed anteriorly and with sharp edge on anterior margin.

##### Description.

**Figure 15.**
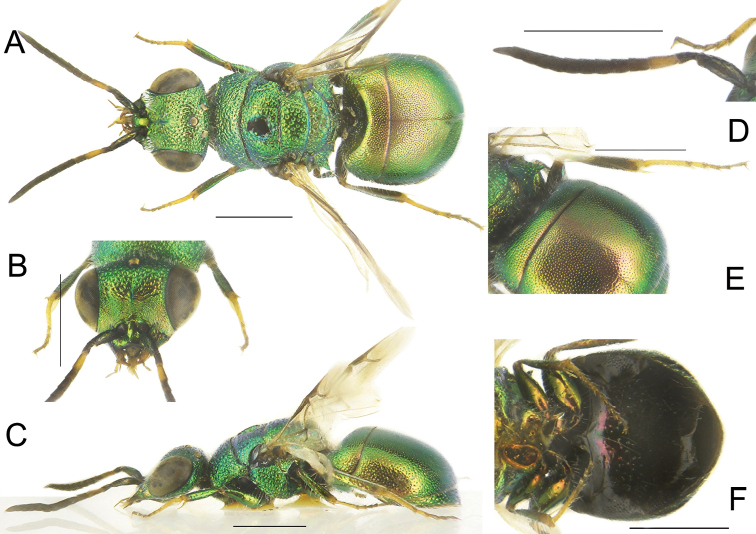
*Hedychridiumvarvarae* sp. nov., female, holotype **A** habitus, dorsal view **B** head, frontal view **C** habitus, lateral view **D** right antenna, lateral view **E** metaleg, right tibia and tarsi, part of metasoma in postero-lateral view **F** metasoma, ventral view. Scale bars: 1 mm.

*Female*. Body length 4.4 mm. *Head.* Face flat, brow slightly convex above scapal basin (Fig. 15A, and B); brow with large, subcontiguous punctures, with wide, polished interspaces; scapal basin finely transversely microridged; face micropunctate laterally and covered by short, appressed, whitish setae; longitudinal mid-line incomplete, distinctly visible from brow to mid-scapal basin only; area in front of anterior ocellus and lateral to posterior ocelli depressed; ocellar triangle isosceles, without ocellar line; malar spaces micropunctate; clypeus apically bordered by semi-circular, brown thickening; mandibles bidentate; mouthparts slightly elongate. Relative length of P:F1:F2:F3 = 1.0:1.3:1.0:0.8; OOL = 1.6 × MOD; POL = 2.0 × MOD; MS = 0.8 × MOD. *Mesosoma.* Pronotum narrowed anteriorly and with sharp edge on anterior margin; coarsely, irregular and uneven-sized punctures, somewhere contiguous to confluent, with polished to corrugated interspaces; mesonotum with wide interspaces, somewhere corrugated; punctures larger at base of mesoscutum and on mesoscutellum, with scattered small punctures; mesopleuron with dense punctures, with small punctures on interspaces; posterior propodeal projections acute, divergent; metatibia flat, with black spot covering large part of its length; metatarsomere 2 as long as metatarsomere 3; pro-, mesopleuron and femora with long whitish setae. *Metasoma*. T1–T3 with minute, dense punctures; punctures denser antero-dorsally on T1–T2; large punctures mixed to minute punctures laterally; S2 with sparse, minute punctures bearing long setae; with violet spot antero-medially, covering less than half segment (Fig. 15F); apical margin of T3 bordered by thin brownish rim. *Colouration.* Head and mesosoma entirely metallic green; metasoma with rosy to bronze reflections (possibly metallic red in nature and when rehydrated in alcohol); scape and pedicel bronze, F1 yellowish; F2 brownish; rest of flagellum brown; tegulae non-metallic brown; femora and tibiae green on outer side, tibial joints largely yellowish; tarsi 1-2 yellowish, tarsi 3-5 brownish; wing membrane clear, nervures light brown.

*Male.* Unknown.

##### Etymology.

The specific epithet *varvara* (feminine, noun in apposition) is dedicated to Varvara M. Proshchalykina (Vladivostok, Russia), for daily support to her father’s research study.

##### Comparative diagnosis.

We describe *Hedychridiumvarvarae* sp. nov. in the *Hedychridiumardens* species group. It is easily separable from all other members of this group by yellowish F1 (black or brown, concolorous with following flagellomeres in the other species) (Fig. 15D); uniform fore body green colouration, including face and propodeum (with contrasting colours at least on face and/or propodeum in other Mongolian species); brow with wide, polished interspaces (usually with dense punctures in other species) (Fig. 15B).

##### Distribution.

Mongolia (Dornogovi).

#### Hedychridium weii

Taxon classificationAnimaliaHymenopteraChrysididae

Rosa, Proshchalykin & Halada sp. nov.

55F68601-CE19-5E44-9FC7-E3E0C6E27DCB

http://zoobank.org/EE06E07F-C9F6-47C5-A087-AB8C0947A322

Figure 16A–G

##### Material examined.

***Holotype*:** ♂, Mongolia: *Dornogovi*, 65 km SE of Chatan-Bulag, steppe, 1020 m alt., 2.VIII.2007, leg. MH (MSNM).

##### Diagnosis.

*Hedychridiumweii* sp. nov. is characterised by elongate shape of the black spot on metatibia; dark colouration; flagellum uniformly black, mesonotal punctures sparse; shape of genital capsule.

##### Description.

**Figure 16.**
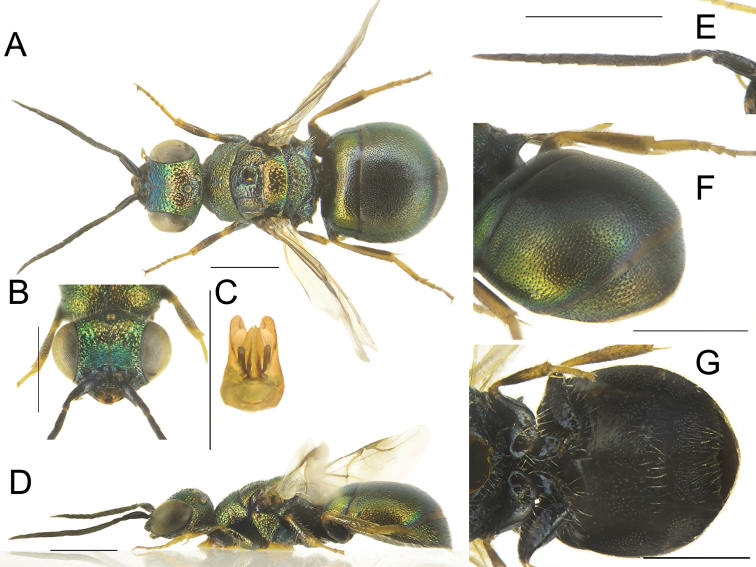
*Hedychridiumweii* sp. nov., male, holotype **A** habitus, dorsal view **B** head, frontal view **C** genital capsule **D** habitus, lateral view **E** right antenna, lateral view **F** metasoma, postero-lateral view **G** metasoma, ventral view. Scale bars: 1 mm.

*Male*. Body length 4.4 mm. *Head.* Face almost flat; scapal basin finely transversely microridged; face between scapal basin and eye with large punctures; longitudinal mid-line complete, extended from brow to clypeus; area in front of anterior ocellus and lateral to posterior ocelli with narrow, deep sulcus; brow with large sized punctures, widely separated by polished interspaces (1 PD) (Fig. 16B); ocellar triangle isosceles, with deep ocellar line; malar spaces impunctate; clypeus apically bordered by narrow, brown thickening; mandibles tridentate. Relative length of P:F1:F2:F3 = 1.0:1.5:1.0:0.8; OOL = 2.0 × MOD; POL = 1.9 × MOD; MS = 0.3 × MOD. *Mesosoma.* Pronotum with coarsely, irregular, uneven-sized punctures, somewhere transversally contiguous to confluent, with polished and corrugated interspaces; mesonotum with contrasting sculpture, with shallow, small and scattered punctures on the anterior half, with larger, denser and confluent punctures on posterior half; mesoscutellum medially with small, sparse punctures and wide polished interspaces (2-3 × PD), laterally and postero-laterally with denser punctures; mesopleuron with dense and large punctures; posterior propodeal projections triangular, divergent; metatibia with elongate dark brown spot covering 4/5 of its length; metatarsomere 2 slightly shorter than metatarsomere 3; pro-, mesopleuron and femora with long whitish setae (1.0–1.5 × MOD). *Metasoma*. T1–T3 with relatively dense, small punctures, anyway not as minute as in *H.erythrosoma* sp. nov.; punctures denser on T1 antero-dorsally; with larger punctures mixed to small punctures laterally; posterior margin of T1–T2 with impunctate, non-metallic black rim, as large as 1.0–1.5 × MOD; S2 without metallic spot, with sparse punctures; S2–S3 with long, whitish setae on posterior margin; apical margin of T3 bordered by thin hyaline rim; genital capsule as in Fig. 16C. *Colouration.* Fore body predominantly coppery, with greenish reflections and bluish propodeum; metasoma greenish, with bronze reflections and non-metallic posterior margin of T1–T2; T1 posteriorly and T2 antero-medially with a large black spot; scape shiny black; pedicel and flagellomeres dull black; tegulae non-metallic brown; femora and tibiae bronze to non-metallic dark brown on outer side, tibial joints largely yellowish; tarsi 1-3 yellowish, tarsi 4-5 brownish; wing membrane hyaline, somehow darkened medially; nervures light brown.

*Female.* Unknown.

##### Etymology.

The specific epithet *weii* (masculine noun in genitive) is dedicated to Na-sen “Nelson” Wei (Guanghzou, China), for his contributions to the study of the Chrysididae of Inner Mongolia and China and his kind support to the studies of the first author.

##### Comparative diagnosis.

We describe *Hedychridiumweii* sp. nov. in the *H.femoratum* species group. It is closely related to *H.femoratum* for its general habitus, yet it is separated by sparser mesonotal punctures, shape of genital capsule (Fig. 16C), elongate shape of the black spot on metatibia (Fig. 16F) and darker mesosoma colouration. It is separated from the other two newly- described species in this species group, *H.splendens* sp. nov. and *H.jacobsi* sp. nov., by flagellomeres black, different genital capsule and different shape of black spots on metatibia.

##### Distribution.

Mongolia (Dornogovi).

### Key to the *Hedychridium* species from Mongolia

**Table:** 

1 Metatarsomere 2 longer or as long as metatarsomere 3 in lateral view	**2**
– Metatarsomere 2 shorter, if slightly shorter, thicker than metatarsomere 3 in lateral view	**7**
2 Body entirely red coloured, at most with greenish-golden propodeum; mouthparts elongate, in lateral view, length from cardo to glossa apex as long as head length; small species (3.0–3.5 mm)	**3 (rhodinum group)**
– Head and mesosoma red to golden-red, usually with contrasting green to blue face and propodeum (exception: *H.varvarae* sp. nov., fully shining green, yet recognisable by F1 yellow); mouthpart unmodified, in lateral view, length from cardo to glossa apex slightly shorter or as long as half head length (exception: *H.frontale* sp. nov., with longer mouthparts); large and robust species (4.0–5.0 mm)	**4 (ardens group)**
3 Subantennal space 1.0 MOD; malar space about 1.0 × MOD; antennae ventrally brown to light brown; mesonotum with wide polished interspaces; tegulae non-metallic; propodeum greenish, slightly contrasting with metanotum; posterior propodeal projections pointed, spiny	***H.gabriellae* Rosa, 2017**
– Subantennal space 1.5 MOD; malar space about 1.4 × MOD; antennae uniformly blackish; mesonotum with dense punctures, without polished interspaces, at most corrugate; tegulae metallic; propodeum shining red, not in contrast with metanotum; posterior propodeal projections blunt	***H.longigena* Rosa, 2017**
4 Female with F1 yellow, contrasting with rest of flagellum; head and mesosoma fully shining green (male unknown)	***H.varvarae* sp. nov.**
– Female with flagellomeres concolorous; at least propodeum green-blue to deep blue	**5**
5 Brow with wide, polished interspaces; scapal basin finely microridged only on lower half, from mid-face to clypeus; clypeal apical margin bordered by thick, slightly arcuate brownish rim 3 × MOD long; head concolorous green, with bluish highlight laterally to clypeus; mouthparts elongate; S2 without metallic spot; legs black to dark purple	***H.frontale* sp. nov.**
– Brow densely punctate; scapal basin mid-line microridged; clypeal apical margin bordered by a short brownish rim not wider than 2 × MOD; head with purplish vertex, greenish brow and blue face; mouthparts short, barely coming out from mandibles; S2 with metallic spot	**6**
6 T2 with large antero-median black spot; posterior margin of metanotum contrasting green to blue; male genital capsule with narrow and evenly round gonocoxa apex	***H.belokobylskiji* Rosa, 2017**
– T2 red coloured, slightly darkened dorsally; posterior margin of metanotum more or less uniform, not distinctly contrasting; male genital capsule with enlarged apex of gonocoxa, angled on inner margin	***H.ardens* (Coquebert, 1801)**
7 Metasoma fully flesh coloured, without metallic reflections in both sexes (in Central Asia other species of the *H.roseum* group may have at least the male with metallic metasoma)	***H.roseum* (Rossi, 1790)**
– Metasoma metallic coloured	**8**
8 Mesoscutum with fine, deep, transversal wrinkles amongst punctures; mesoscutellum with fine, deep, longitudinal wrinkles amongst punctures; species dorsally entirely bronze coloured (Fig. 14A)	***H.striatum* sp. nov.**
– Mesonotum with polished or punctate interspaces among punctures, without wrinkles	**9**
9 F1 yellowish, contrasting with scape, pedicel and rest of flagellum	**10**
– Flagellomeres uniformly coloured, dark brown to black	**11**
10 Long F1 (l/w = 2.3 in female, 3.0 in male) (Fig. 11D); sharp transverse ridges covering almost all face in frontal view; mesonotum with dense and deep punctures, with narrow polished interspaces and small punctures amongst large ones (Fig. 11A); metasoma with dense and deep punctures	***H.jacobsi* sp. nov.**
– Short F1 (l/w = 2.0 in female) (Fig. 13D); scapal finely microridged only on lower half, from mid- face to clypeus; mesonotum with sparse and shallow punctures, with wide polished interspaces amongst punctures (Fig. 13A); metasoma with sparse and shallow punctures	***H.splendens* sp. nov.**
11 Tibiae yellowish or tibiae widely yellowish distally and medially light brown, with or without slight metallic reflections; metasoma ventrally without metallic spot or with only small trace on S2	**12**
– Tibiae dark with metallic reflections, at least with a reduced distal area non-metallic brownish; tarsi brownish, tarsomere 1 lighter; metasoma ventrally with metallic spot	**13**
12 Scutellum with dense punctures (Fig. 9A and D); metanotum blue, contrasting with coppery scutellum (Fig. 9A); inner side of metatibia with small black spot (Fig. 9F); male genital capsule as in Fig. 9C	***H.femoratum* (Dahlbom, 1854)**
– Scutellum with small, spaced punctures and wide polished interspaces (Fig. 16A); metanotum coppery, not contrasting with scutellum (Fig. 16A); inner side of metatibia with elongate black spot (Fig. 16D and F); male genital capsule as in Fig. 16C	***H.weii* sp. nov.**
13 Metanotum blue, contrasting with mesonotum red, golden-red or coppery (variability observed in prepared specimens, due to collecting methods and dehydration) (Fig. 6D)	***H.asianum* Linsenmaier, 1997**
– Metanotum and scutellum concolourous red (Figs 8A, B and 14A)	**14**
14 Propodeum blue, contrasting red metanotum (Fig. 12A); pronotum laterally with a violet transversal stripe (Fig. 12D); metatarsomere 2 slender and as long as metatarsomere 3; head, legs with short setae (1.0–1.5 MOD); medium sized species (4.0–5.0 mm); genital capsule as in fig. 14C and D in Rosa et al. (2017a)	***H.leleji* Rosa, 2017**
– Propodeum red, concolorous with metanotum (Fig. 6A and B); pronotum red; metatarsomere 2 shorter and thicker than metatarsomere 3; head and legs with long setae (1.5–2.0 MOD); large species (5.0–6.0 mm); genital capsule as in Fig. 8F	***H.erythrosoma* sp. nov.**

**Genus *Holopyga* Dahlbom, 1845**

*Holopyga* Dahlbom, 1845: 4. Type species: *Holopygaamoenula* Dahlbom, 1845, by subsequent designation of Ashmead 1902: 227.

#### Holopyga lucida

Taxon classificationAnimaliaHymenopteraChrysididae

(Lepeletier, 1806)

5F5FECC3-856F-5971-849A-116243B3DC56

Hedychrum lucidum Lepeletier, 1806: 122. Syntypes; France (Paris, Turin ?).

##### Material examined.

Mongolia: *Tuv*, 1 ♀, 50 km N of Ulaanbataar, river E of Mandal, 1180 m alt., 8–13.VII.2007, leg. MK (PRC).

##### Distribution.

*Mongolia (Tuv). *Holopygalucida* is distributed from Europe to Eastern Siberia (Rosa et al. 2019).

##### Remarks.

The Mongolian specimen has narrower scapal basin, more arcuate beneath brow compared with European specimens; however, the ratio between head width and inter-ocular distance is almost equal. Moreover, the punctation of the Mongolian specimen is without wide, polished interspaces, whereas European specimens have mesonotal punctures sparser, with shining intervals. We did not observe additional differences and, therefore, we identify this specimen as *H.lucida*, waiting for the examination of more material.

#### Holopyga similis

Taxon classificationAnimaliaHymenopteraChrysididae

Mocsáry, 1889

E4A9BF0D-9504-5571-B7F6-8E45516AC671

Holopyga (Holopyga) similis Mocsáry, 1889: 130. Lectotype ♀, designated by Móczár 1964: 439; Hungary (HNHM) (examined).

##### Material examined.

Mongolia: *Tuv*, 1 ♀, Khangaun Mts, 5 km N of Khunt, 21.VII.2005, leg. PT (PTC).

##### Remarks.

*Holopygasimilis* was synonymised by Bischoff (1913) with H.gloriosavar.chrysonota Förster, 1853. Rosa et al. (2020) pointed out that *H.chrysonota* is a different species and *H.similis* is the first available name for *H.chrysonota* sensu Linsenmaier (1959, 1987).

##### Distribution.

*Mongolia (Tuv). South-east Europe to Caucasus, Turkey and Israel (Rosa et al. 2019).

#### Holopyga tyrneri

Taxon classificationAnimaliaHymenopteraChrysididae

Rosa, Proshchalykin & Halada sp. nov.

0A304344-6105-5F60-875F-FA4D2A55E7F1

http://zoobank.org/1273F58D-CE9D-4374-8608-7FC5B6B0A7A6

**Figure 17.**
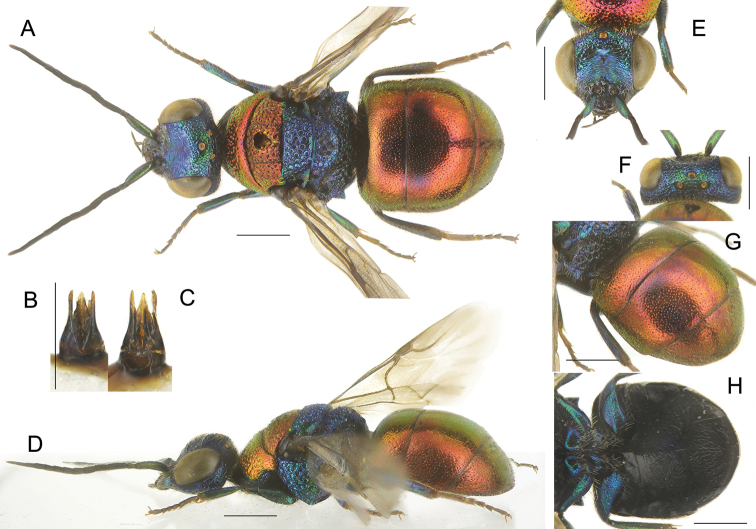
*Holopygatyrneri* sp. nov., male, holotype **A** habitus, dorsal view **B** genital capsule, dorsal view **C** genital capsule, ventral view **D** habitus, lateral view **E** head, frontal view **F** head, dorsal view **G** metasoma, postero-lateral view **H** metasoma, ventral view. Scale bars: 1 mm.

**Figure 18.**
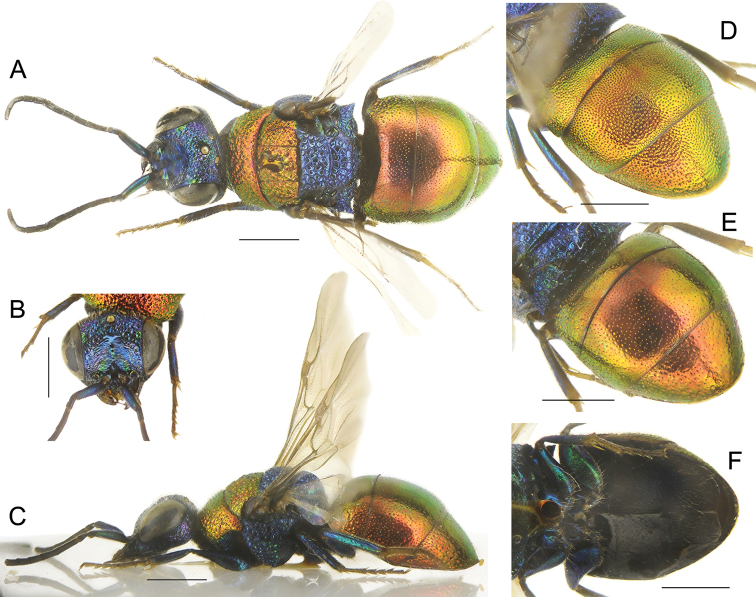
*Holopygatyrneri* sp. nov., female, paratype (**A–C, E, F**) and *H.similis* Mocsáry, female from (from Italy) (**D**) **A** habitus, dorsal view **B** head, frontal view **C** habitus, lateral view **D** metasoma, postero-lateral view **E** metasoma, postero-lateral view **F** metasoma, ventral view. Scale bars: 1 mm.

Figures 17A–H , 18A–C, E, F

##### Material examined.

***Holotype*:** ♂, Mongolia, *Zavkhan*, 40 km SW of Uliastay, dunes, 18.VII.2005, leg. JH (MSNM). ***Paratypes***: 4 ♀♀, 2 ♂♂, *Zavkhan*, 40 km SW of Uliastay, dunes, 18.VII.2005, leg. JH (MHC); 1 ♀, 1 ♂, same date and locality of holotype (MHC); 4 ♀♀, *Arkhangai*, 25 km NE of Tsetserleg, 47°38'N; 101°45'E, 23.VII.2004, leg. JH (MHC); 1 ♀, *Bulgan*, 137 km NE of Aravaykheer, 47°20'N; 103°40.5'E, 1250 m alt., 26.VII.2004, leg. JH (PRC); 2 ♀♀, *Dornod*, 50 km SW of Choibalsan, 960 m alt., 25.VII.2007, leg. JH (MHC); 1 ♀, 100 km W of Choibalsan, 820 m alt., 23.VII.2007, leg. MH (MHC); 1 ♂, 2 km SE Khuvsgol, 5.VIII. 2007, leg. PT (PTC); 1 ♂, *Sukhbaatar*, 200 km SSE of Baruun-Urt, Moltsoy Els, 1250 m alt., 27.VII.2007, leg. MH (MHC); 3 ♀♀, 100 km SSW of Baruun-Urt, 1100 m alt., 30.VII.2007, leg. MH (MHC).

##### Diagnosis.

*Holopygatyrneri* sp. nov. is characterised by metasoma with noticeably scattered punctures, with shallow punctures on terga and relatively dense punctures on S2; head and mesosoma deep blue, with pronotum and mesoscutum flame red; metasoma red flame to golden-red.

##### Description.

*Male*. Body length 6.0–6.9 mm (holotype 6.9 mm). *Head.* Brow and vertex with irregularly-sized punctures, with narrow, polished interspaces; punctures on face between scapal basin and eye larger; face shallowly hollowed; scapal basin transversally microridged, polished below brow, glabrous; genae with coarsely, irregular and confluent punctures; mandibles bidentate; ocellar triangle isosceles, with deep ocellar line connecting posterior ocelli. Relative length of P:F1:F2:F3 = 1.0:2.0:1.2:1.2; OOL = 2.2 × MOD; POL = 2.3 × MOD; MS = 0.5 × MOD. *Mesosoma*. Pronotum with deep, irregularly-sized punctures mixed with small dots on interspaces; posterior margin with small dots only; mesoscutum with shallow, large punctures (0.5 × up to 1.0 × MOD on basal half), with scattered dots anteriorly on narrow interspaces; punctures dense and subcontiguous basally; mesoscutellum with larger (1.0 × MOD), irregular and dense punctures with narrow, polished interspaces; notauli and parapsidal lines deep, as fine lines; with large antero- and postero-median area polished and with shallow small dots; mesopleuron with dense, large punctures, irregularly confluent along posterior margin; metascutellum with dense large punctures, as large as those on mesoscutellum, with narrower interspaces; propodeal posterior projections small, subparallel, pointing slightly outwards. Legs and wings unmodified compared with similar species. *Metasoma.* Metasomal terga with even and minute punctures, equally spaced dorsally, 2-3 PD apart (Fig. 17E); with some denser, larger punctures on T1 antero-laterally; apical margin of T3 with narrow brownish rim; S2–S3 with dense punctures (Fig. 17F). *Colouration.* Head and mesosoma deep blue, with pronotum and mesoscutum flame red; metasoma red flame to golden-red; scape green, pedicel and flagellum black; clypeus non-metallic brown; legs metallic green to blue with brownish tarsi; metasomal sterna black without metallic reflections; forewing hyaline, slightly darkened medially.

*Female.* Body length 6.0–7.0 mm. Similar to male in habitus and colour pattern and with dimorphic T3, acutely arcuate (Fig. 18E). Flagellum I distinctly longer l/w = 3.5 (l/w 2.5 in male); posterior propodeal projections more divergent and acute; metasoma with more scattered, minute punctures.

##### Etymology.

The specific epithet *tyrneri* (masculine noun in genitive) is dedicated to Pavel Tyrner (Litvínov, Czech Republic), who collected and provided data from Mongolia and for his precious contribution to the knowledge of the European Chrysididae.

##### Comparative diagnosis.

*Holopygatyrneri* sp. nov. is closely related to *Holopygasimilis* Mocsáry, 1889 [= *H.chrysonota* sensu Linsenmaier (1959)] for habitus and same colour pattern in both sexes. It can be immediately separated by metasoma with noticeably scattered and shallow punctures (Fig. 18E) compared with *H.similis* (Fig. 18D) and by different genital capsule. The female also resembles *Holopygachrysonota* (Förster, 1853) [= *H.ignicollis* sensu Linsenmaier (1959)] by colour pattern; however, it can be separated by metasoma with scattered, shallow punctures and by denser punctures on S2 (vs. scattered with only few dots in *H.chrysonota*). The male of *Holopygachrysonota* is differently coloured, with mesosoma green, pronotum and mesoscutum usually lighter.

##### Distribution.

Mongolia (Arkhangai, Bulgan, Dornod, Sukhbaatar, Zavkhan).

**Genus *Philoctetes* Abeille de Perrin, 1879**

*Philoctetes* Abeille de Perrin, 1879: 27. Type species: *Holopygacicatrix* Abeille de Perrin, 1879 [= *Philoctetesmicans* (Klug, 1835)], by subsequent designation of Ashmead 1902: 228.

#### Philoctetes boreki

Taxon classificationAnimaliaHymenopteraChrysididae

Rosa, Proshchalykin & Halada sp. nov.

72BFA052-B1DB-532E-9A54-6BC0E13405A4

http://zoobank.org/24E38692-5029-46D1-8996-3D1488D682AA

Figure 19A–G

##### Material examined.

***Holotype*:** ♂, Mongolia, *Tuv*, Khangaun Mts, 5 km N of Khunt, 20.VII.2005, leg. JH (MSNM).

##### Diagnosis.

*Philoctetesboreki* sp. nov. is characterised by greenish-blue body colour and metanotal projection, more or less projecting over propodeum; flattened body; shallow punctuation and long, blackish erect setae.

##### Description.

**Figure 19.**
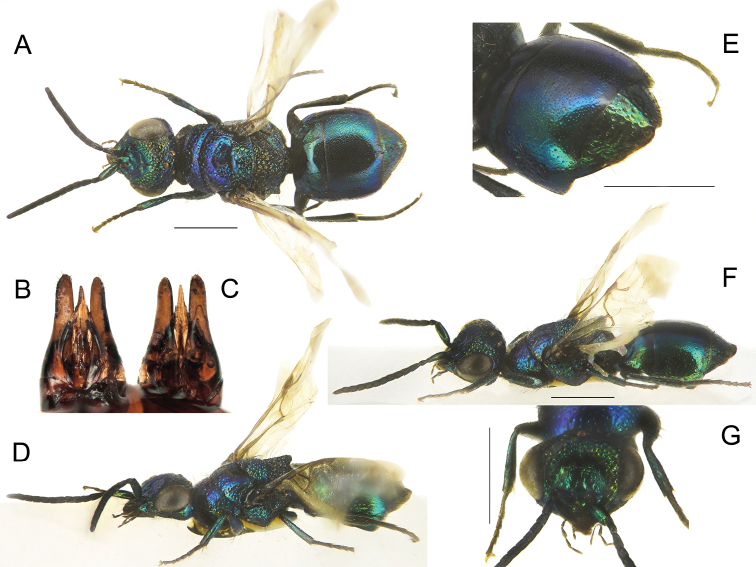
*Philoctetesboreki* sp. nov., male, holotype **A** habitus, dorsal view **B** genital capsule, ventral view **C** genital capsule, ventral view, **D** habitus, dorso-lateral view **E** metasoma, postero-lateral view **F** habitus, lateral view **G** head, frontal view. Scale bars: 1 mm.

*Male*. Body length 4.8 mm. *Head.* Brow, vertex, face between eye and scapal basin with dense, large-sized punctures (0.5–0.7 MOD) (Fig. 19A); with two impunctate areas laterad posterior ocelli; scapal basin asetose, deep and hemicircular (Fig. 19G), with irregular wrinkles following the scapal basin contour; gena with small punctures; genal carina sharp, not bisecting MS; ocellar triangle isosceles, postocellar line indistinct; anterior margin of clypeus straight, thickened, non-metallic brown. Relative length of P:F1:F2:F3 = 1.0:1.5:1.1:1.0; OOL = 3.3 × MOD; POL = 2.7 × MOD; MS = 0.6 × MOD; genae, brow and temples with elongate, thick setae (1.5–2.0 × MOD). *Mesosoma.* Pronotum with sparse, shallow and small punctures, smaller than punctures on head; interspaces polished and wide (up to 3 PD); mesoscutum with small, shallow punctures mostly clumped along notauli and parapsidal lines (Fig. 19A); punctures at base of mesoscutum larger; notauli line deep and narrow; parapsidal line deep and complete; mesoscutellum with dense, large punctures (up to 1 × MOD), antero-medially with polished area; metascutellum longer than mesoscutellum, mucronate, with elongate and triangular lamella apically rounded; mesopleuron with irregular-sized punctures; posterior propodeal projections short and blunt; mesosoma, including femora, with black, elongate and thick setae. *Metasoma*. T1 antero-medially polished, with scattered dots on posterior margin, laterally with double punctation with larger, deep punctures mixed with small, sparse dots; T2 with small, even and uniformly scattered punctures dorsally; with double punctation laterally, as on T1; T3 with irregular deep and larger punctures and few scattered dots; lateral edge of T3 slightly sinuous medially; apical margin of T3 bordered by non-metallic brown rim; apical notch deep, triangular (Fig. 19E); T3 with long (2.0 MOD), black and thick setae. *Colouration.* Body deep blue with light blue to green areas on face, metascutellum, mesopleuron and mesosoma laterally; scape green, pedicel and flagellum black; tegulae dark brown; forewing slightly hyaline; meso- and metafemur unusually dark brown; T3 covered with long, erect, thick setae.

*Female.* Unknown.

##### Etymology.

The specific epithet *boreki* (masculine noun in genitive) is dedicated to Borek Halada (České Budějovice, Czech Republic), son of Marek, for his precious contribution in the organisation of the present article.

##### Comparative diagnosis.

*Philoctetesboreki* sp. nov. is related to a few other high altitude Alpine and Central Asian species. They all share some morphological features, such as flattened body, shallow punctuation and long, blackish erect setae (Rosa et al. 2017b). It shares with the Alpine *Philoctetesputoni* (du Buysson, 1892) and *P.helveticus* (Linsenmaier, 1959) greenish-blue body colour and metanotal projection, more or less projecting over propodeum. *P.boreki* sp. nov. is separated from these species by distribution of black setae, mostly focused on the last visible tergum and different shape of metanotal plate (Rosa et al. 2017b). Central Asian species belonging to this group are *P.elongatus* (Semenov-Tian-Shanskij & Nikol’skaya, 1954) (from Tajikistan), *P.hirsutus* (Semenov-Tian-Shanskij, 1932) (Kyrgyzstan and Uzbekistan) and *P.hirtus* (Semenov-Tian-Shanskij, 1932) (Kyrgyzstan). *P.boreki* sp. nov. is separated from these Central Asian species by mucronate metascutellum (vs. metanotum conical, without distinct mucronate projection) and by *P.hirtus* for green-blue body colour (vs. metasoma metallic red). Another Central Asian species, *P.lyubae*, shares with *P.boreki* sp. nov. body uniformly coloured, although green to golden-green and elongate metascutellar plate, yet the habitus is normally shaped, not flattened, with short, whitish setae.

##### Distribution.

Mongolia (Tuv).

## Conclusions

The Mongolian Chrysididae fauna is largely unknown and a first, preliminary list was recently published by Rosa et al. (2020) with a total of 90 species in 18 genera. In this article, we list another eight species *Chrysisinclinata* Linsenmaier, 1959, *C.martinella* du Buysson, 1900, *C.speciosa* Radoszkowski, 1877, *Euchroeuspurpuratus* (Fabricius, 1787), *Holopygalucida* (Lepeletier, 1806), *H.similis* Mocsáry, 1889, *Hedychridiumfemoratum* (Dahlbom, 1854), *H.leleji* Rosa, 2017 and excluded two from the previous checklist: *Hedychridiumcupreum* (Dahlbom, 1845) and *H.propodeale* Rosa, 2017. We also describe eleven new species which increase the number of known Mongolian species to 107 species in 18 genera. The high number of new species, described in this article and the new records for the countries listed in this and in the previous paper (Rosa et al. 2020), show how poor the current knowledge is of the Mongolian and the Central Asian fauna in general. In fact, the new records extend eastwards the distribution of several species by thousands of kilometres (e.g. *Chrysisinclinata*, *Hedychridiumfemoratum*). Due to this largely incomplete knowledge, the richness of the Mongolian chrysidid fauna cannot be assessed with confidence yet, underlining the need to improve research projects in this country and in the Central Asian Countries as well.

## Supplementary Material

XML Treatment for Chrysis inclinata

XML Treatment for Chrysis martinella

XML Treatment for Chrysis mocsaryi

XML Treatment for Chrysis speciosa

XML Treatment for Chrysis strakai

XML Treatment for Chrysis woodi

XML Treatment for Euchroeus purpuratus

XML Treatment for Hedychridium belokobylskiji

XML Treatment for Hedychridium erythrosoma

XML Treatment for Hedychridium femoratum

XML Treatment for Hedychridium frontale

XML Treatment for Hedychridium jacobsi

XML Treatment for Hedychridium leleji

XML Treatment for Hedychridium splendens

XML Treatment for Hedychridium striatum

XML Treatment for Hedychridium varvarae

XML Treatment for Hedychridium weii

XML Treatment for Holopyga lucida

XML Treatment for Holopyga similis

XML Treatment for Holopyga tyrneri

XML Treatment for Philoctetes boreki
